# A structural dissection of protein–RNA interactions based on different RNA base areas of interfaces†

**DOI:** 10.1039/c8ra00598b

**Published:** 2018-03-16

**Authors:** Wen Hu, Liu Qin, Menglong Li, Xuemei Pu, Yanzhi Guo

**Affiliations:** College of Chemistry, Sichuan University Chengdu 610064 People's Republic of China yzguo@scu.edu.cn liml@scu.edu.cn +86-028-85412290 +86-028-85412290

## Abstract

Protein–RNA interactions are very common cellular processes, but the mechanisms of interactions are not fully understood, mainly due to the complicated RNA structures. By the elaborate investigation on RNA structures of protein–RNA complexes, it was firstly found in this paper that RNAs in these complexes could be clearly classified into three classes (high, medium and low) based on the different levels of *P*_base_ (the percentage of base area buried in the RNA interface). In view of the three RNA classes, more detailed analyses on protein–RNA interactions were comprehensively performed from various aspects, including interface area, structure, composition and interaction force, so as to achieve a deeper understanding of the recognition specificity for the three classes of protein–RNA interactions. According to our classification strategy, the three complex classes have significant differences in terms of almost all properties. Complexes in the high class have short and extended RNA structures and behave like protein–ssDNA interactions. Their hydrogen bonds and hydrophobic interactions are strong. For complexes in low class, their RNA structures are mainly double-stranded, like protein–dsDNA interactions, and electrostatic interactions frequently occur. The complexes in medium class have the longest RNA chains and largest average interface area. Meanwhile, they do not show any preference for the interaction force. On average, in terms of composition, secondary structures and intermolecular physicochemical properties, significant feature preferences can be observed in high and low complexes, but no highly specific features are found for medium complexes. We found that our proposed *P*_base_ is an important parameter which can be used as a new determinant to distinguish protein–RNA complexes. For high and low complexes, we can more easily understand the specificity of the recognition process from the interface features than for medium complexes. In the future, medium complexes should be our research focus to further structurally analyze from more feature aspects. Overall, this study may contribute to further understanding of the mechanism of protein–RNA interactions on a more detailed level.

## Abstract 2

Qualitative and quantitative measurements of the influence of structure and composition of RNA interfaces on protein–RNA interactions.

## Graphical abstract

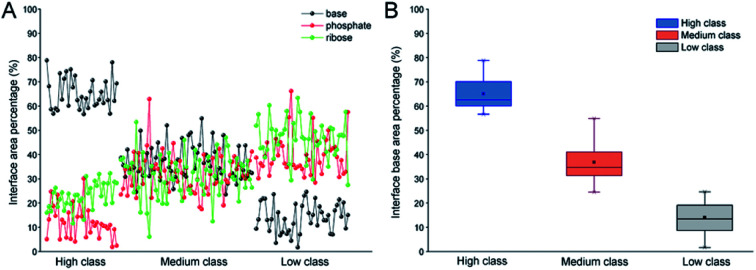

## 1. Introduction

Nucleic acids, including DNAs (deoxyribonucleic acids) and RNAs (ribonucleic acids), always function through interactions with proteins. Such interactions play crucial roles in a wide variety of biological processes. Protein–DNA interactions (PDIs) are essential for DNA transcription, packaging, replication and repair.^1–3^ Protein–RNA interactions (PRIs) are indispensable for the regulation of gene expression, protein synthesis, RNA splicing and post-transcriptional control.^4–7^ It is urgent and quite meaningful to precisely understand the recognition mechanisms of PDIs and PRIs. Since PDIs have been widely reviewed before PRIs, insufficient structure data limit the further development of research on PRIs.^8,9^ With recent advances in biological technology, the number of available PRI structures are increasing, which provides an opportunity to launch a structure-based analysis on the principles governing the interactions between proteins and RNAs. These research studies on PRIs mainly include the construction of PRI databases,^10,11^ sequential or structural comparisons between PRIs and PDIs,^12,13^ prediction of RNA-binding sites,^14–17^ and structural dissection of protein–RNA interfaces.^18–20^

In recent years, much attention has been paid to examining the general interface properties of protein–RNA complexes.^21–28^ Bahadur *et al.*^22^ analyzed PRIs in terms of interface size, composition, polar interactions and atomic packing and found electrostatic complementation, base recognition and shape complementarity on the interfaces of PRIs. By investigating the preferred RNA structural states in protein-binding regions, Gupta *et al.*^24^ observed strong preferences for both RNA bases and RNA structural states in protein–RNA interactions, indicating their mutual importance in protein recognition. The work of Iwakiri *et al.*^26^ suggests that nucleotide bases in the RNA loop are flipped out and form hydrogen bonds with the proteins, and different protein surface shapes prefer different RNA base-pairing properties. The most recent report by Barik *et al.*^28^ compared the structural, geometric and physicochemical properties of interfaces involved in protein–RNA, protein–DNA and protein–protein interactions. The result indicates that H-bonds, salt bridges and stacking interactions play significant roles in stabilizing PRI interfaces. Despite the great progress in PRI research, the structural mechanism underlying PRIs is still not fully understood, owing to the amazing diversity of RNA structures. Compared to double-stranded DNAs, RNA molecules display a much wider variety of conformations and shapes.^29^ Moreover, a nucleotide is composed of the negatively charged phosphate, neutral ribose and polar base. As we know, the properties of different RNA interfaces commonly determine the different interacting modes of RNAs with proteins. Therefore, the initial aim of our work is to qualitatively and even quantitatively measure the influence of the structure and composition of RNA interfaces on protein–RNA interactions.

Firstly, the relationship between composition and structure of the RNA interface was explored. We collected a non-redundant dataset of 137 X-ray structures of protein–RNA complexes and analyzed the contents of phosphate, ribose and base on each RNA interface. It was interestingly found that the three RNA groups show obvious composition differences among these 137 complexes, but the most significant difference is observed in terms of base composition. *P*_base_ (the percentage of base buried area in the RNA interface) can be as high as over 80% or lower than 10%. According to the different values of *P*_base_, the 137 RNA interface structures can be clearly clustered into three classes (high, medium and low). Then, in order to understand the recognition process specificity of the three RNA classes, a comprehensive feature analysis was implemented on interface structures, intermolecular physicochemical properties and interface forces. Systematic comparisons among the three classes of complexes suggest that their interfaces are obviously different in terms of most features, which shows that the classification of RNA interfaces based on *P*_base_ is reasonable. We demonstrate that the interface area contributed by the RNA base group could strongly influence protein recognition and binding, indicating that it can be used as a new determinant to distinguish different types of protein–RNA complexes. Thus, our analysis may contribute to understanding the specificity of the recognition process and the identification of protein–RNA binding sites on a deeper level.

## 2. Materials and methods

### 2.1 Dataset of the protein–RNA complexes

Protein–RNA complex structures were obtained from Protein Data Bank (PDB) database^30^ (Feb 2014) with X-ray structures and resolution better than 3.0 Å as criteria. In the present study, we only extracted those protein–RNA complexes containing proteins with at least 20 amino acid residues and RNA molecules with at least 5 nucleotides (nt). Some PDB chains containing only C_α_ atoms were excluded from our dataset. Moreover, some ribosomal subunits and viral protein–RNA complexes were also ignored in our dataset because these complexes often contain a large number of amino acid residues on interfaces and most of the RNA interfaces on proteins have not been determined, which could lead to population bias. For each protein–RNA complex, we chose a representative and stable biological assembly using the PDBePISA tool.^31^ Thus the entire dataset consists of 487 complexes (listed in ESI Table S1†). In order to remove redundancy, we used CDHIT^32^ to align RNA and protein sequences from the dataset. Sequence identity threshold of 30% for proteins and 90% for RNAs was respectively used. The final non-redundant dataset includes 137 complexes, which are detailed in ESI Table S2.†

### 2.2 Definition of interface

In this paper, the software NACCESS^33^ was used to calculate the solvent accessible surface (ASA) values. The interface area of a protein–RNA complex was calculated using the web-based tool PRince,^34^ which uses NACCESS with a probe radius of 1.4 Å and default group radii. The size of a protein–RNA interface area (IA) was estimated by subtracting the ASA of the complex from the sum of the ASAs of the individual subunits, as shown in eqn (1):

1 IA = ASA_protein_ + ASA_RNA_ − ASA_complex_

Here, the interface atoms are referred to as those that lose solvent accessibility and contribute to IA in a complex. In previous studies,^14,19,35^ a residue with at least one interface atom was always defined as the interface residue. Based on eqn (1), the *P*_ribose_, *P*_phosphate_ and *P*_base_ were calculated using the following equations:2
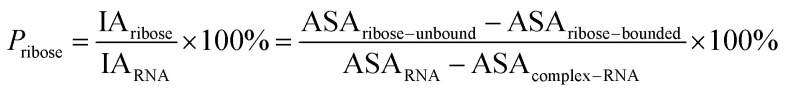
3
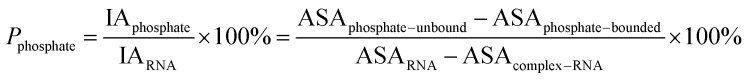
4
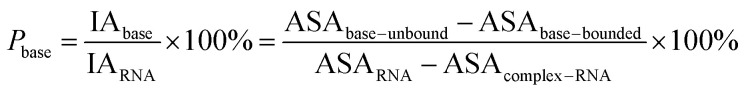


### 2.3 Definition of RNA structure

We used RNA view^36^ to identify and classify the types of nucleotide pairs. In our study, paired nucleotides are defined as any of 12 families of base pairs,^37^ and the remaining nucleotides are considered unpaired. Then, we calculated the *R*_pair_, which indicates the ratio of the number of paired nucleotides to all nucleotides. *R*_pair_ represents the degree of pairing of RNA. In a protein–RNA complex, a smaller *R*_pair_ indicates more single-stranded regions in the RNA.

### 2.4 Interface properties

Here, six important interface properties were calculated to reveal the structural foundations of different complexes. They are the interface area (IA), the ratio of interface area to surface area (*R*_*i*/*s*_), amino acid composition (AAC), amino acid propensity (AAP), secondary structure composition (SSC) and secondary structure propensity (SSP).

IA is defined as the total ASA decrease of one protein and one RNA upon interaction, and it reflects the size of the interfaces (eqn (1)). *R*_*i*/*s*_ is the ratio of the interface area to the rest of the complex surface area (eqn (5)):5
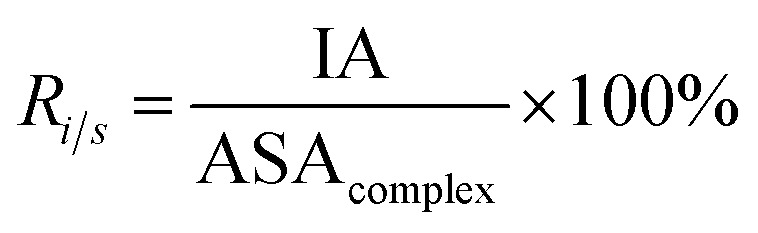


AAC is defined as the occurrence frequencies of the 20 standard amino acids in the interface residue sets, expressed as:6
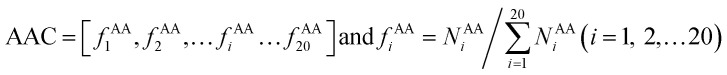
where *f*^AA^_*i*_ represents the frequency that amino acid type *i* contributes to the protein–RNA interface residue sets. *N*^AA^_*i*_ is the number of the amino acid type *i*.

The AAP shows the enrichment or depletion of each type of amino acid in the interface as compared to the entire protein surface.^38^ The AAP can be calculated as:

7  AAP = [*P*^AA^_1_,  *P*^AA^_2_, … *P*^AA^_*i*_ … *P*^AA^_20_]  and *P*^AA^_*i*_ = ln(*f*^AA^_*i*_/ *f*^AA, SURF^_*i*_)

where *f*^AA,  SURF^_*i*_ is the frequency of the *i*-th amino acid in the protein surface.

The program STRIDE^39^ was employed to assign the protein secondary structures. Six secondary structure types were considered, including α-helix, β-strand, turn, coil, bridge and 3_10_-helix. Turn, coil, bridge and 3_10_-helix were together deemed as the non-regular (NR) regions. The SSC is defined as follows:8
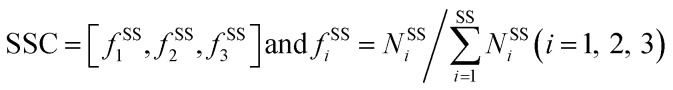
where *f*^ss^_*i*_ is the occurrence frequency of a particular secondary structure type in the interface residue sets, and *N*^SS^_*i*_ is the corresponding number of the secondary structure type. The SSP is calculated as follows:

9 SSP = [*P*^SS^_1_,   *P*^SS^_2_,   *P*^SS^_3_] and  *P*^SS^_*i*_ = ln(*f*^SS^_*i*_/* f*^SS,SURF^_*i*_)   

where *f*^SS, SURF^_*i*_ is the occurrence frequency of a particular secondary structure type in a protein surface.

### 2.5 Interface force

Here, five kinds of noncovalent interactions were considered, including hydrogen bonds, electrostatic forces, van der Waals contacts, hydrophobic interactions and stacking interactions. Hydrogen bonds (H-bonds) at protein–RNA interfaces were calculated using the software HBPLUS,^40^ and positively charged electrostatic patches on protein surfaces were obtained through BindUP.^41^ For each protein–RNA complex, we calculated the percent overlap between the largest electrostatic positive patches on protein surfaces and the RNA-binding interfaces of each chain (*P*_e_) and the mean *P*_e_ (*P̄*_e_):^42^10
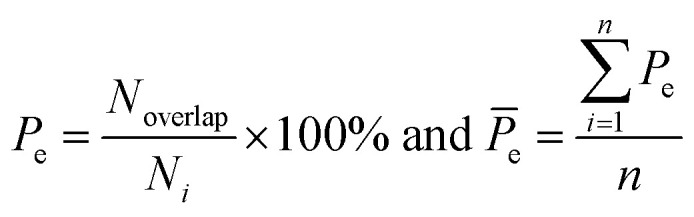
where *N*_overlap_ is the number of the overlapped residues between positive patch and the binding interface, *N*_*i*_ is the number of interface residues, and *n* is the number of amino acid chains in each complex. *P̄*_e_ reflects the electrostatic property of the interface. In addition, the exact electrostatic energy of each complex was calculated by the MM/GBSA approach^43^ using MMPBSA.py tools^44^ in the Amber16 package.^45^

van der Waals contacts, hydrophobic interactions and stacking interactions were measured by the program ENTANGLE.^46^ van der Waals contacts are denoted as the sum of the van der Waals radii of the two atoms plus a maximum distance (defined ≤ 1.0 Å). Stacking interactions are defined as the π–π interactions that can occur between the side chains of Tyr, Trp, Phe, His and the bases. Moreover, we also considered the π–π and π–cation stacking of Arg through its guanidinium moiety onto nucleosides. Hydrophobic interactions are deemed as non-polar atoms that are ≤5.0 Å apart. We calculated the percent overlap between the hydrophobic interface and the RNA-binding interfaces of each chain (*P*_h_) and the average *P*_h_ (*P̄*_h_):11
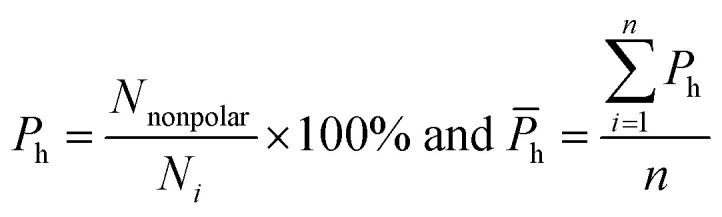
where *N*_nonpolar_ is the number of overlapped residues between the hydrophobic interface and binding interface; *P̄*_h_ can reflect the hydrophobic property of the interface.

## 3. Results and discussion

### 3.1 Statistical analysis of protein–RNA complex data

**Fig. 1:**
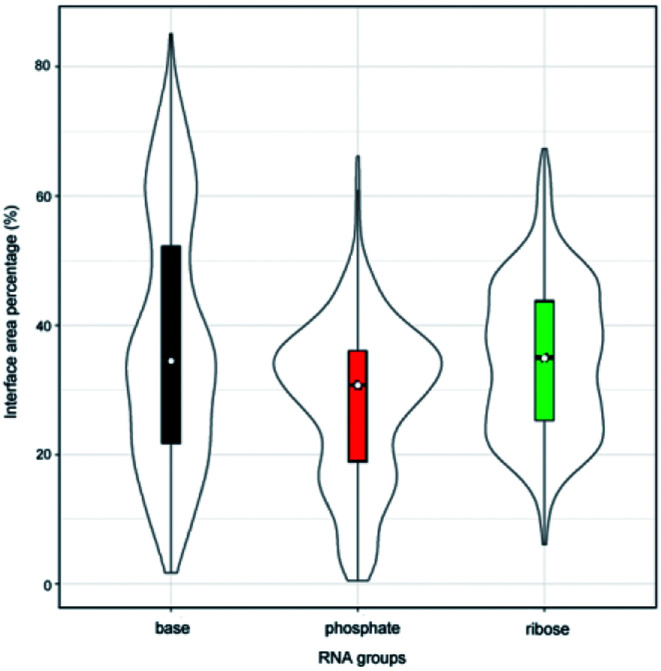
Violin plot combining the box plot and density trace for *P*_ribose_, *P*_phosphate_ and *P*_base_ in the initial dataset.

Originally, we calculated the content of the ribose, phosphate and base buried in the RNA interface area, designated *P*_ribose_, *P*_phosphate_ and *P*_base_, respectively, for the initial dataset (487 complexes, ESI Table S1†), including non-redundant and all remaining redundant complexes. We found that 98% of all complexes have *P*_phosphate_ values of <50% and 92%, and of which the *P*_ribose_ values were lower than 50%. By contrast, the *P*_base_ values show significant differences among all complexes and are widely distributed between 0% and 80%. This can be seen from the violin plot shown in Fig. 1. The ribose and phosphate moieties are the non-specific parts of the RNA molecules, so the differences between RNA molecules are not significant in terms of *P*_ribose_ and *P*_phosphate_. The *P*_base_ represents the interface area contributed by base groups, which are specific to RNA molecules. Thus, we could consider whether the interface area contributed by RNA base residues can be used as a new standard for distinguishing protein–RNA complexes.

**Fig. 2:**
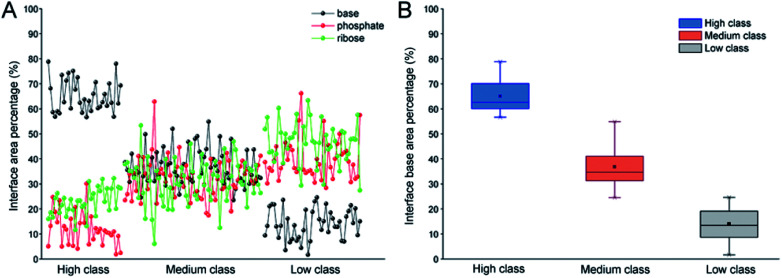
Distribution of *P*_ribose_, *P*_phosphate_ and *P*_base_ in 137 non-redundant protein–RNA complexes. (A) The percentage of *P*_ribose_, *P*_phosphate_ and *P*_base_ in different types of protein–RNA complexes. (B) The box plot for *P*_base_ in each class.

In order to validate the reasonability of this classification, we used the 137 non-redundant complexes for more detailed calculations (ESI Table S2†). Fig. 2A shows the distribution of *P*_ribose_, *P*_phosphate_ and *P*_base_ in 137 non-redundant protein–RNA complexes. We could easily observe the significant differences between these complexes based on *P*_base_ values and classified them into three classes (high, medium and low). As a result, high includes 33 complexes with the average *P*_base_ value of 65% and standard deviation (SD) of 6.5%. Medium comprises 61 complexes (*P*_base_ = 37% ± 6.2%), and low consists of 43 complexes (*P*_base_ = 14% ± 7.2%, Fig. 2B).

**Fig. 3:**
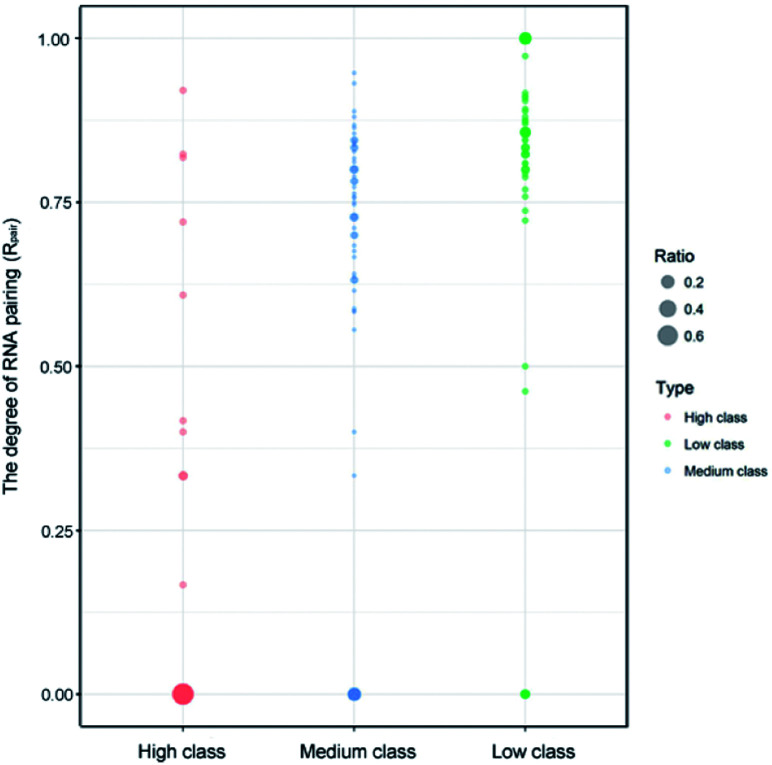
Bubble chart of *R*_pair_ in the three classes. The bigger bubble and the deeper color indicate higher frequency of *R*_pair_ in each class.

Indeed, previous studies have shown that protein interactions with the RNA ribose-phosphate backbone are more common than interactions with the bases.^19,22,24,47,48^ So, the number of complexes in high class is lower than that in medium and low. We also counted the numbers of different structures and types of RNAs in the three classes. Fig. 3 shows the distribution of *R*_pair_ in different types of protein–RNA interactions. It suggests that most RNA molecules (23/33) in high are single stranded in structure with *R*_pair_ = 0, while those in low are double stranded in structure (31/43), with *R*_pair_ value greater than 0.8. In medium, RNA structure is more complicated because of the widely distributed *R*_pair_ values. For RNA types, we consider the five common types (ssRNA, dsRNA, tRNA, rRNA and mRNA), and other RNAs were deemed as ‘other’ type. The detailed information is listed in ESI Table S3.† The most important RNA types are mRNA, tRNA and dsRNA in high, medium and low, respectively. Therefore, we can conclude that both the RNA structures and types display obvious differences among the complexes in the three classes.

A diagrammatic structure analysis was also performed on the complexes in the three classes, and Fig. S1† gives the 3D structures of three representative samples (PDB ID: 3QJJ, 1F7U and 3VYY). We found that most RNAs are in single-stranded form when interacting with proteins in the high class, which exposes the base groups of RNAs on the interfaces. So, we can explain why *P*_base_ values are high in the high class. Meanwhile, those in low mostly use their stem or double-stranded regions to bind to proteins, so the *P*_base_ values are lower than others. In medium, RNAs can have both single and double-stranded states to interact with proteins, so the *P*_base_ values are medium.

### 3.2 Interface property analysis of different classes of protein–RNA complexes

Here, we calculated the interface properties for the protein–RNA complexes in three classes. Table 1 gives the average values of different properties.

**Table 1:** Average properties of the protein–RNA interfaces

Interface	Protein–RNA^a^			
	All	High	Medium	Low
Number of complexes	137	33	61	43
IA (Å^2^)	3192 ± 1822	2729	3808	2673
IA_protein_ (Å^2^)	1526 ± 881	1255	1833	1299
IA_RNA_ (Å^2^)	1666 ± 945	1474	1975	1375

**Number of**				
Amino acids	54 ± 31	46	65	45
Nucleotides	20 ± 11	12	24	20
Protein atoms	178 ± 103	154	213	147
RNA atoms	172 ± 99	147	205	146

**IA (Å** ^**2**^ **) per**				
Amino acid	28.3 ± 4.8	27.1	28.3	29.3
Nucleotide	93.4 ± 36.9	124.6	90.3	73.7

**Surface area buried ratio (%)**				
Complex	14.0 ± 5.9	17.5	13.1	12.5
Protein	8.8 ± 4.8	9.3	8.0	9.6
Nucleic acid	28.5 ± 16.3	36.7	27.1	24.2

a Data are expressed as mean ± standard deviation (SD).

#### 3.2.1 IA and *R*_*i*/*s*_

**Fig. 4:**
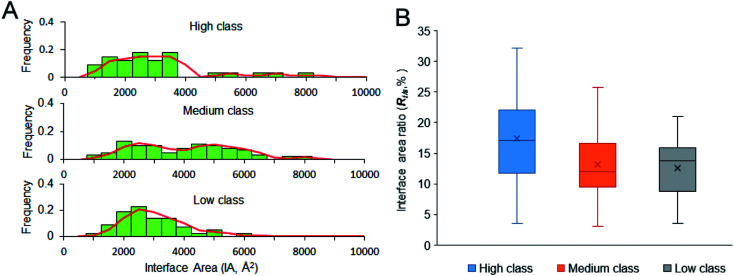
Size of the interfaces and interface area ratio of our dataset. (A) The frequency histogram of interface area size. (B) The box plot for interface area ratio of the three classes.

IA is deemed an important property for macromolecular interactions.^49,50^ From Table 1, the average IA is 2729 Å^2^, 3808 Å^2^ and 2673 Å^2^ in high, medium and low, respectively. For the non-redundant dataset, the average IA is 3192 Å^2^, which is contributed by 54 amino acids and 20 nucleotides. Fig. 4A shows the frequency histogram reflecting the distribution of IA in each class. In medium class, the IA values are found in a wide range, from 900 Å^2^ to 8000 Å^2^, except for two complexes (PDB ID: 2GIC and 4JNG; IA = 10 344 Å^2^ and 11 308 Å^2^) due to the four or five protein chains on their interfaces. About 50% of medium complexes have IA > 4000 Å^2^; however, the IA sizes of high and low complexes are from 2000 to 4000 Å^2^. In high and low class, the distribution of IA has a peak at 2500 Å^2^. In addition to the same peak at 2500 Å^2^, the distribution of IA in medium class has another peak at 5000 Å^2^, which is consistent with the previous report, giving two broad peaks at 2000 Å^2^ and at 4800 Å^2^ for whole protein–RNA interfaces.^28^ This result indicates that the second peak is mainly contributed by the medium class. So, in terms of IA, complexes in high and low are similar to each other, but they are obviously different from those in medium (*P* < 0.05).

A stable interface needs not only a large IA but also high *R*_*i*/*s*_.^51,52^Fig. 4B displays the box plot for *R*_*i*/*s*_ in different types of protein–RNA interactions. It reveals that complexes in high class have the highest average *R*_*i*/*s*_, with the average values of *R*_*i*/*s*_ declining from high to medium and then to low, showing a different trend from the observation on IA in Fig. 4A. In Fig. 4A, complexes in medium give the largest average IA. This result may indicate that the protein and RNA surfaces in high class are more likely to be involved in the interfaces when they contact with each other to form complexes. Moreover, short and extended RNA structures of high complexes make them more conducive to interact with proteins. The complexes in medium class have both large interface area and large surface area because of the large molecular weight. Lastly, complexes in low may have more unstable interfaces than the two other classes because of the low IA and *R*_*i*/*s*_. So, in terms of *R*_*i*/*s*_, complexes in high are significantly different from those in medium and low (*P* < 0.01).

#### 3.2.2 Number of interface atoms, residues or nucleotides

**Fig. 5:**
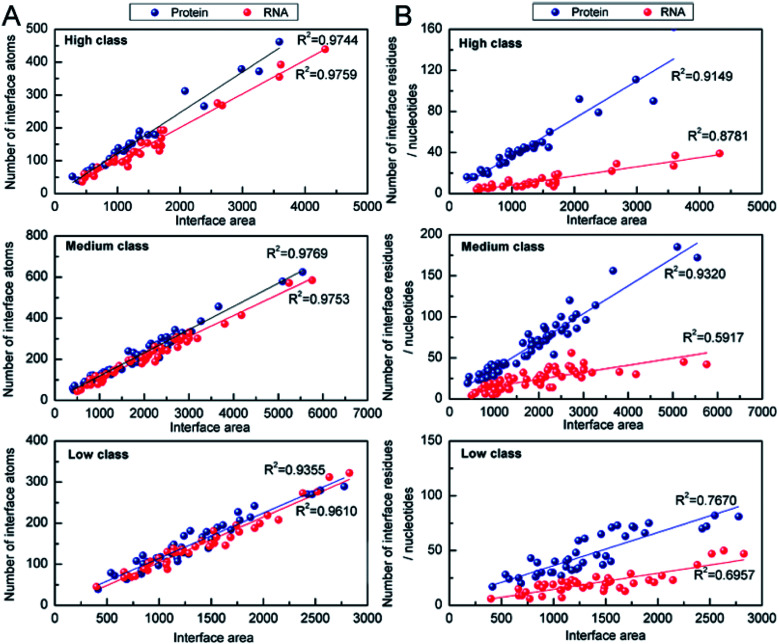
Analysis of the correlation coefficients between *N*_atoms_ (or *N*_residues_/*N*_nucleotides_) and interface area. (A) Number of interface atoms against the interface area for the three classes. (B) Interface residues or nucleotides against the interface area for the three classes.

The number of interface atoms and residues/nucleotides on the protein and RNA interface are respectively shown in Table 1. The IA for each interface nucleotide is 125 Å^2^, 90 Å^2^ and 74 Å^2^ in high, medium and low, respectively. Compared with the protein–DNA complexes,^53^ the complexes in high behave like protein and single-stranded DNA complexes, with the IA of 130 Å^2^ for each interface nucleotide. However, those in low behave like protein and double-stranded DNA complexes with the IA of 68 Å^2^. Then, we calculated the correlation coefficients (*R*^2^) between the number of interface atoms and IA for each class. The results in Fig. 5A show very good linear correlation, with the *R*^2^ values in high, medium and low all much higher than 0.90 for both the interface RNA atoms and the interface protein atoms. This result is consistent with the previous studies,^22,28,54^ which have confirmed that whether the complexes are in the high class, medium class or low class, the correlation coefficients between the number of interface atoms and interface area are high in both the protein and the RNA components.

The correlation between the number of interface residues/nucleotides and IA was investigated for each class. We found that the values of *R*^2^ in Fig. 5B are always lower than the *R*^2^ in Fig. 5A. On the protein side, we obtained a satisfactory *R*^2^ of 0.91, 0.93 and 0.76 in high, medium and low class, respectively. On the RNA side, medium class yields the minimum *R*^2^ (*R*^2^ = 0.59), while *R*^2^ is 0.88 and 0.70 in high and low class, respectively. This result may be due to the more complicated RNA structures of medium complexes. Previous studies have reported that the linear correlation between IA and the number of interface nucleotides is low, with *R*^2^ of 0.67.^22,28^ From our results, we can explain that the complexes in medium may be key samples for this mediocre correlation.

#### 3.2.3 AAC, AAP, SSC and SSP

**Fig. 6:**
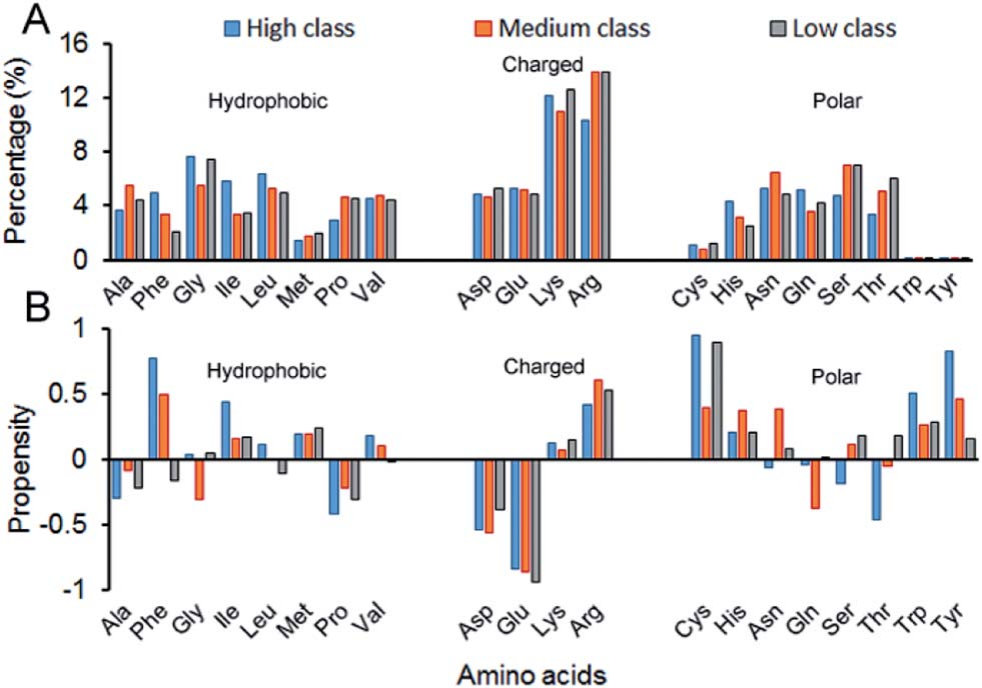
Amino acid composition and propensity of each class. (A) The average percentage of 20 amino acids in the interface of each class. (B) The average propensity of 20 amino acids in each class.

Here, we calculated the composition (AAC) and propensity (AAP) of the 20 amino acids on the interface residues in each class (Fig. 6). Twenty standard amino acids are classified into three categories according to their physicochemical properties: Ala, Phe, Gly, Ile, Leu, Met, Pro and Val belong to hydrophobic residues; Asp, Glu, Lys and Arg are deemed charged residues; and Cys, His, Asn, Gln, Ser, Thr, Trp and Tyr are polar residues. From Fig. 6A, for all the 137 structures, the total composition of positively charged amino acids on interfaces is maximum. The reason is obviously clear: RNA phosphate groups are negatively charged, so they prefer to interact with positively charged amino acids.^13,19,22,29^ Though in all classes, the AACs of positively charged amino acids are all relatively high, their preferred residues are different. In high, the largest contribution comes from Lys, while it is Arg in medium and low. Then, we calculated the total percentage of both Lys and Arg, and we found that the total percentage increases from high to medium, and then to low (ESI Table S4†). The reason may be that complexes in low have the lowest *P*_base_ on the interfaces, which promotes the phosphate backbones to interact with proteins. The percentage of other residues contributing to the interface is also high except for the three polar residues of Cys, Thr and Tyr, which agrees with previous observations.^22^Fig. 6B shows the relative propensity of 20 amino acids. It can be seen that the interfaces are far from the protein surface in high class, especially for the hydrophobic residues. This result indicates that hydrophobic residues may contribute significantly to binding RNA for complexes in high class. Moreover, for the entire dataset, the negatively charged amino acids are more likely to appear on the protein surfaces than on the interfaces. Similar observations have also been found in previous studies.^13,55^

**Fig. 7:**
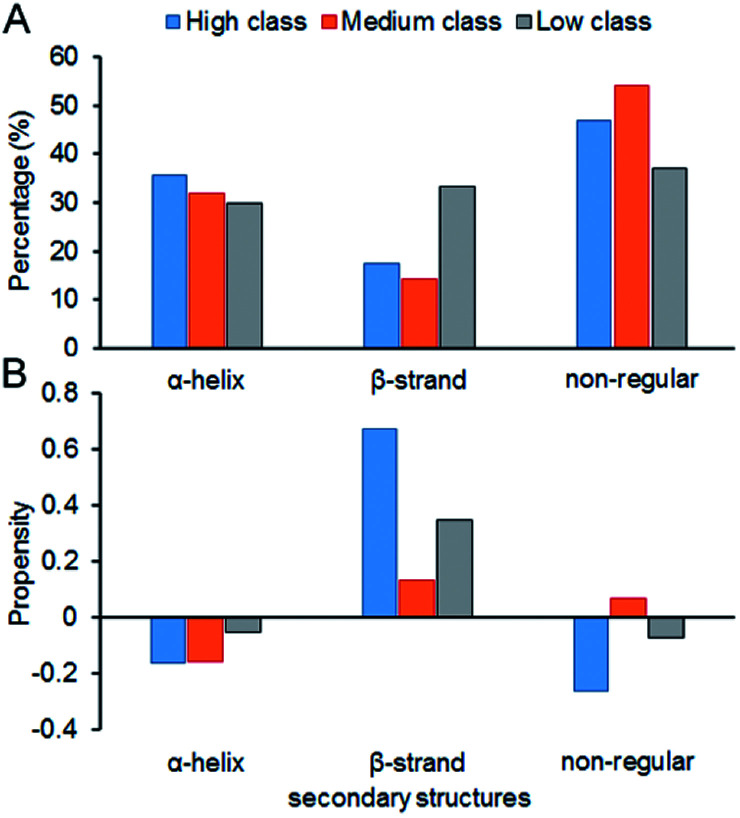
Protein secondary structure composition and propensity of each class. (A) The average percentage of secondary structures in the interface of each class. (B) The average propensity of secondary structures in each class.

Fig. 7 shows the composition (SSC) and propensity (SSP) of the three types of secondary structures in each class, including α-helix, β-strand and the non-regular regions. Similar to that reported by Gupta and Gribskov,^24^ the non-regular elements are the primary protein interface structural state (Fig. 7A). Moreover, medium class yields the maximum percentage of non-regular regions. This may be due to the more complicated structures of RNAs in medium, so it is more difficult for them to bind the regular structures of proteins, such as α-helix and β-strand. Therefore, the structures of binding proteins in medium tend to be non-regular. In Fig. 7B, we can easily obtain the same conclusion that β-strands are preferred on protein–RNA interfaces, but α-helix does not show obvious propensity.^55,56^ In high class, this phenomenon is more obvious. The reason may be that β-strand is less likely to interface with the RNA backbone,^24^ which gives the RNA base a greater chance to bind with β-strand. The details on the composition and propensity of secondary structures in each class are listed in ESI Table S5.†

### 3.3 Interaction force analysis on the different classes of protein–RNA complexes

#### 3.3.1 Hydrogen bonds

For all the complex data, there is a total of 2853 hydrogen bond contacts, and the average number of hydrogen bonds that equal that of the protein–DNA complexes is 22.^22,28^ Medium class has the largest average number of hydrogen bonds, while low class has the least. We know that the number of H-bonds on interfaces is closely related to the size of interfaces.^55^ For each complex, we calculated the H-bond density, which reflects the strength of H-bond on interfaces. Similar with the trend of *R*_*i*/*s*_ in Fig. 4B, although the average number of hydrogen bonds is highest in medium class, the density of hydrogen bonds is low because the size of interface is large. For high class, H-bond density is highest, which means the hydrogen bonds are strongest.

We also counted the frequency of all the chemical components for H-bonds in each class (Table 2). On the protein side, the frequency of main chains increases from high to medium and then to low, probably because in high, the RNA structures are more extended and they more easily interact with protein backbones. The main chain nitrogen has been proven to be more frequently found than the main chain oxygen in protein–RNA H-bonds,^28^ which can also be obviously observed from the medium- and low-class complexes in our study, but not those in high. The reason is also the influence of different *P*_base_ values for the three classes; the base tends to form hydrogen bonds with protein main chain oxygen atoms, while the phosphate tends to be with nitrogen atoms.^22^ In the side chain involved in hydrogen bonds, the content of charged groups is nearly twice that of neutral groups in all protein–RNA interactions. However, in protein–DNA complexes, the contents of charged and neutral groups nearly equal each other.^47^ On the RNA side, the contribution of phosphate and ribose to protein–RNA H-bonds is 61%, which is less than that in protein–DNA H-bonds (76%). The frequencies of different RNA bases involved in H-bonds are also different. U (14%) and G (10%) are more frequently found than A (7%) and C (8%). Interestingly, only in low class is the frequency of G (7%) larger than the frequency of U (2%), and this phenomenon has also been found in protein–DNA H-bonds.^27,47^ Overall, these results suggest that the complexes in high have the strongest hydrogen bonds, and H-bonds in low are similar to the protein–DNA H-bonds, both on the protein side and the RNA side.

**Table 2:** Chemical compositions of H-bonds

H-bonds	All	High	Medium	Low
Total number	2933	675	1529	729
Number per interface^a^	22	20	26	17

**Protein chemical group (%)** b				
Main chain O	12	18	10	9
Main chain N	15	16	15	14
Side chain groups				
Charged	46	42	49	47
Neutral	27	23	26	31

**Nucleic acid chemical group (%)** b				
Phosphate	41	17	50	57
Sugar	20	16	17	26
Base	39	66	33	17
Guanine	10	15	8	7
Adenine	7	12	7	3
Cytosine	8	10	8	5
Uracil/thymine	14	29	11	2

a Average number of H-bonds per interface.

b Percentage of 2933 protein–RNA H-bonds contributed by the protein or nucleic acid chemical group.

#### 3.3.2 Electrostatic force

**Fig. 8:**
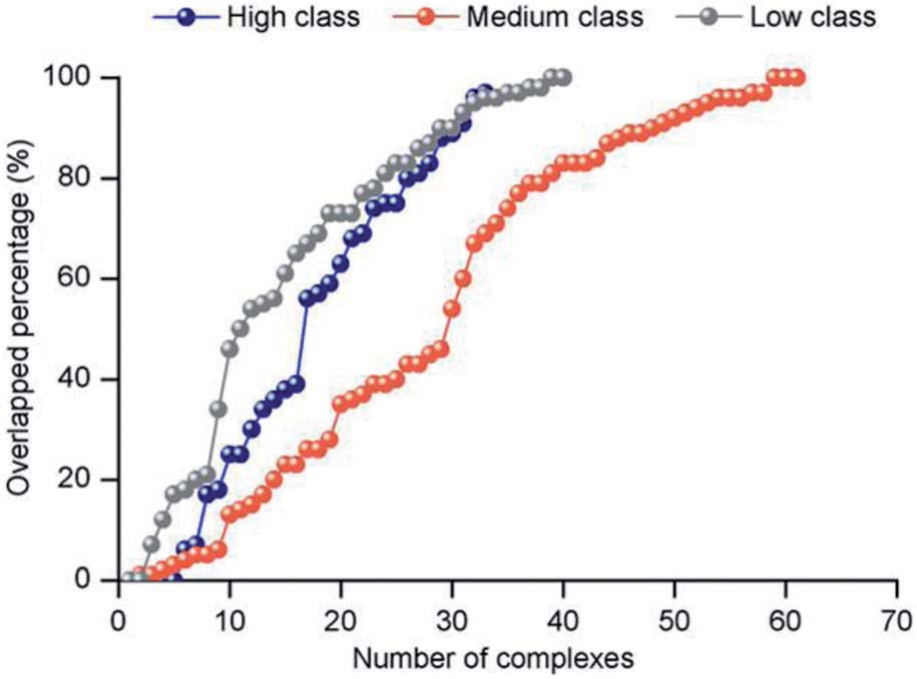
The distribution of the overlapped percentage between the largest electrostatic positive patches and interface.

The electrostatic force plays an important role in bio-macromolecule interactions, especially during the “lure” step.^21^ We studied the electrostatic force in each class from two aspects. Firstly, large positive patches are deemed an important property of protein surfaces, and they are usually considered a sign of binding interfaces.^57–60^ To investigate the electrostatic properties of the interface, we calculated the percent overlap between the largest electrostatic positive patches on the protein surfaces and the binding interfaces in each class (Fig. 8). For all the non-redundant 137 structures, the average percent overlap is 56%. By contrast, the average percent overlap between patches and the interfaces is 75% in protein–DNA complexes.^26^ This result can be attributed to the negatively charged phosphate groups of double-stranded DNAs having more chance to electrostatically interact with proteins. Moreover, from 0% to 100%, the distribution of percent overlap ranges from dense to sparse, then dense. In high class, the average percent overlap is minimum (47.8%), while in low class, it is maximum (64.9%), and in medium, it is also low (55.2%). Moreover, a similar trend is found on the average electrostatic energy of the three classes (ESI Table S2†). The average electrostatic energy is −2038.1 kcal mol^−1^, −3052.3 kcal mol^−1^ and −5050.7 kcal mol^−1^ in high, medium and low class, respectively. Thus, our result demonstrates that the complexes in low have the strongest electrostatic energy, and the size of the interface involved in electrostatic interaction has significant difference among the three classes of complexes. The work of Nilofer *et al.*^61^ has shown a poor correlation between interface area and electrostatic energy in the protein–protein interface. Our conclusion is consistent with this, since the correlation coefficient between IA and electrostatic energy is only 0.06 for the dataset.

#### 3.3.3 Stacking interactions

The stacking interaction refers to attractive and noncovalent interactions between aromatic rings. These interactions are important in protein–RNA interfaces.^25,46^ We calculated stacking interactions for each protein–RNA complex. Table 3 lists the number of stacking interactions in the three different interface classes. In total, 250 stacking interactions were found in our 137 protein–RNA interfaces, and the average number of stacking interactions per complex in the entire dataset is about 2. The contribution of stacking interactions by the interfaces in high, medium and low class is 57.2%, 37.2% and 5.6%, respectively. The differences among them are significant (*P* < 0.05). Obviously, all the stacking interactions in high are stronger than those in medium and low. However, the contribution of residues and bases involved in stacking interactions is similar among the three classes. On the protein side, the side chains of Arg and Tyr are involved in more than 60% of all stacking interactions; however, the number of Trp involved in stacking interactions is least. Similar observations have also been found in the previous studies.^28^ On the RNA side, U and A are more frequently found than G and C in all stacking interactions. The number of stacking interactions contributed by purines (A, G) and pyrimidines (C, U/T) almost equal each other at 48% and 52%, respectively.

**Table 3:** Number of stacking interactions

	High class				Medium class				Low class				All			
Residues	A	G	C	U	A	G	C	U	A	G	C	U	A	G	C	U
Phe	3	11	1	14	3	3	1	14	0	0	1	0	6	14	3	28
Tyr	9	10	0	29	3	4	0	4	0	0	1	0	12	14	1	33
His	6	9	0	8	3	0	3	3	0	0	0	0	9	9	3	11
Trp	3	0	0	0	3	1	1	3	0	1	1	0	6	2	2	2
Arg	21	3	6	10	10	5	10	19	2	7	1	0	32	15	17	29
**Average** a	**4**				**2**				**0**				**2**			

a Average number of stacking interactions per complex in each class.

Overall, our results are consistent with previous studies using the same data set ,^25,28,46^ and the contribution of residues and bases is similar in each class. Further, our results show significant differences in the strength of stacking interactions among the three classes. The complexes in high have the strongest stacking interactions, while in low complexes, they are very weak and RNAs may interact with the protein by other types of interaction forces.

#### 3.3.4 van der Waals forces

The van der Waals contact is a basic intermolecular force which is closely related to the atomic spatial distance. We analyzed the number of the van der Waals forces in each class. Here, the main role of the van der Waals force is to stabilize the macromolecular structure, and the strength only depends on the atomic distances.^61^ Our results suggest that there is no obvious difference among the three classes in terms of the van der Waals density on interfaces. Compared with H-bonds, it is easy to see that the van der Waals contact is a nonspecific force for each complex, so the preference of the RNA backbone and bases involved in van der Waals interactions is relatively weak.^55,62^

#### 3.3.5 Hydrophobic interaction

**Fig. 9:**
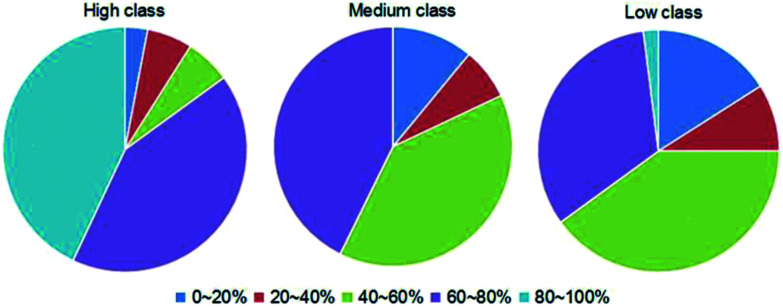
Pie chart of the distribution of the five categories of *P̄*_h_ (the percent overlap between hydrophobic interface and RNA-binding interfaces) in each class. The first to the fifth categories are designated as the *P̄*_h_ ranges of 0–20%, 20–40%, 40–60%, 60–80% and 80–100%, respectively, as indicated by dark blue, red, green, dark purple and cyan, respectively.

To comprehensively explore the differences in interaction forces among our three classes, we finally analyzed the strength of hydrophobic interactions in each class. The hydrophobic interaction is one of the fundamental forces in the protein–nucleic acid interface.^23,63^ We calculated the percent overlap between the hydrophobic and RNA-binding interfaces (*P̄*_h_) of each complex. The values are distributed between 0% and 100%, and the details are listed in ESI Table S2.† Then, we divided *P̄*_h_ into five categories by the values, which are 0–20%, 20–40%, 40–60%, 60–80% and 80–100%, respectively. The distributions of the five categories of *P̄*_h_ in each class are shown in Fig. 9. For almost all complexes of high class (30/33), more than half of the interface residues are involved in hydrophobic interactions. Moreover, 15 complexes in high class have more than 80% overlap between binding interface and hydrophobic interface. However, no complexes were found in medium to have such a high percent overlap, and only 1 was found in low class. These results suggest that hydrophobic interactions play a much more important role in high class complexes than in medium and low, since hydrophobic interactions are defined as non-polar atoms that are ≤5 Å apart in the ENTANGLE package.^46,62^ In contrast to the phosphates and the bases, due to the presence of the 2′OH, it is harder for the ribose in RNA to form hydrophobic interactions.^27^

## 4. Conclusions

Protein–RNA interactions play important roles in a wide variety of biological processes.^4–7^ The different structures or conformations of RNA molecules may influence the binding protein sites.^23,28^ Moreover, the base is a special part of RNA and is frequently involved in important interactions.^22,24,26,48^ In the study, to qualitatively and further quantitatively measure the influence of the RNA composition on protein–RNA interactions, we firstly proposed a new standard to distinguish protein–RNA complexes based on the percentage of the base area buried in the RNA interface area. As a result, a dataset of 137 protein–RNA complexes was divided into three classes (high, medium and low). We comprehensive analyzed the properties of protein–RNA interactions, including interface compositions, interface structures, intermolecular physicochemical properties and interface forces, and also analyzed the difference between the three class complexes as well as compared them with protein–DNA interfaces reported in previous research.^12,13,47,53^ The results are clear: complexes in high class have the shortest RNAs and the RNA structures are mainly single stranded, which facilitates the interaction of the flipped or exposed base group with proteins. These complexes behave like protein–ssDNA interactions. Among the five types of interactions, H-bonding and hydrophobic interaction are strong, while the electrostatic interaction is weak. The complexes in medium have the longest RNAs and the largest interface area; however, the interface ratio is the smallest. The linear correlation between IA and number of interface nucleotides is the worst because of the irregular and more complicated RNA structures. Meanwhile, the interaction forces do not show any preference. In low class, the interface area distribution is similar with that of high class. The RNA structures are mainly double-stranded and behave like protein–dsDNA interactions. The interface propensity of Lys is high. Compared to high class, the electrostatic interaction is strong, while stacking and hydrophobic interactions are very weak. According to our classification strategy, the three classes of complexes have significant differences in terms of almost all properties. Unlike the high or low complexes, we cannot easily understand the specificity of the recognition processes of medium complexes based on the interface features. Therefore, we would pay more attention to the medium complexes in the future. Moreover, it is necessary to develop specific predictors for complexes in different classes, and different classes of protein–RNA complexes should be studied individually. Our study proves that the size of interface area contributed by the RNA base group can highly impact the properties of RNA–binding proteins and may play an important role in understanding the mechanism of protein–RNA interactions.

## Conflicts of interest

There are no conflicts to declare.

## Supplementary Material

RA-008-C8RA00598B-s001
