# Supplementary material for: A structural dissection of protein–RNA interactions based on different RNA base areas of interfaces
Source: RSC Adv. 2018 Mar 16;8(19):10582–92. doi: 10.1039/c8ra00598b (PMC9078961; doi:10.1039/c8ra00598b)
Supplement: RA-008-C8RA00598B-s001 [file RA-008-C8RA00598B-s001.pdf]

Electronic Supplementary Material (ESI) for RSC Advances.
This journal is © The Royal Society of Chemistry 2018

**Tabel S1.** The detailed value of $P_{ribose}$, $P_{phosphate}$ and $P_{base}$ in the 487 protein-RNA complexes.

| PDB ID | Protein chain | RNA chain | $P_{base}$ | $P_{phosphate}$ | $P_{ribose}$ | PDB ID | Protein Chain | RNA Chain | $P_{base}$ | $P_{phosphate}$ | $P_{ribose}$ |
|---|---|---|---|---|---|---|---|---|---|---|---|
| 1a9n | B | Q | 62.10 | 15.97 | 21.93 | 1asy | A | R | 40.42 | 30.80 | 28.79 |
| 1asz* | A | R | 39.95 | 26.32 | 33.73 | 1av6 | A | B | 14.87 | 43.47 | 41.66 |
| 1b23 | P | R | 24.05 | 28.99 | 46.96 | 1b7f* | A | P | 65.84 | 11.34 | 22.81 |
| 1c0a | A | B | 34.93 | 30.62 | 34.45 | 1c9s | LMNOPQRSTUV | W | 77.80 | 0.48 | 21.73 |
| 1dfu* | P | MN | 24.63 | 39.95 | 35.42 | 1di2* | AB | CDEG | 9.52 | 32.87 | 57.61 |
| 1dk1 | A | B | 13.89 | 34.60 | 51.51 | 1drz | A | B | 62.94 | 19.38 | 17.68 |
| 1dul | A | B | 37.96 | 13.49 | 48.55 | 1e7k* | A | C | 56.67 | 30.14 | 13.18 |
| 1ec6* | A | D | 63.12 | 5.84 | 31.04 | 1efw* | A | C | 32.79 | 37.45 | 29.75 |
| 1euy | A | B | 35.34 | 26.47 | 38.18 | 1exd | A | B | 35.16 | 25.66 | 39.18 |
| 1f7u* | A | B | 38.62 | 23.49 | 37.89 | 1f7v | A | B | 38.77 | 22.93 | 38.30 |
| 1f7y* | A | B | 9.39 | 38.72 | 51.88 | 1feu | A | BC | 21.66 | 35.30 | 43.04 |
| 1ffy | A | T | 19.58 | 31.86 | 48.55 | 1fxl | A | B | 57.93 | 20.47 | 21.60 |
| 1g1x* | ABC | DE | 15.86 | 37.75 | 46.39 | 1g2e | A | B | 59.22 | 17.71 | 23.07 |
| 1g59 | A | B | 23.52 | 32.01 | 44.47 | 1gax* | A | C | 37.34 | 23.56 | 39.10 |
| 1gtf* | LMNOPQRST | W | 78.82 | 5.10 | 16.06 | 1gtn | LMNOPQRSTUV | W | 75.09 | 4.96 | 19.95 |
| 1gtr* | A | B | 35.55 | 25.89 | 38.56 | 1gts | A | B | 36.00 | 25.54 | 38.47 |
| 1h3e* | A | B | 30.77 | 33.98 | 35.25 | 1h4q | AB | T | 53.20 | 15.96 | 30.83 |
| 1h4s | AB | T | 53.44 | 16.14 | 30.42 | 1hc8 | A | C | 13.71 | 30.94 | 55.35 |
| 1hq1 | A | B | 33.74 | 17.70 | 48.56 | 1i6u* | A | C | 11.62 | 31.50 | 56.88 |
| 1il2* | A | C | 35.03 | 31.91 | 33.06 | 1ivs | A | C | 37.29 | 24.06 | 38.65 |
| 1j1u | A | B | 52.12 | 13.00 | 34.88 | 1jbr | AB | CDF | 51.48 | 27.93 | 20.60 |
| 1jbs | A | C | 43.43 | 35.10 | 21.47 | 1jbt | A | C | 43.61 | 34.45 | 21.94 |
| 1jid* | A | B | 15.10 | 55.16 | 29.74 | 1k8w | A | B | 31.95 | 38.88 | 29.17 |
| 1kq2* | ABHIKM | R | 59.11 | 16.90 | 23.99 | 1kuq | A | B | 15.99 | 30.00 | 54.00 |
| 1l9a* | A | B | 15.07 | 57.47 | 27.45 | 1lng | A | B | 23.99 | 48.59 | 27.42 |
| 1m5k | C | B | 63.53 | 15.63 | 20.83 | 1m5o | C | B | 64.24 | 14.76 | 21.00 |
| 1m5p | C | B | 62.44 | 15.82 | 21.74 | 1m5v | C | B | 64.08 | 15.38 | 20.54 |
| 1m8v* | ABCDEFGHI | OPQRSTU | 65.97 | 7.87 | 26.16 | 1m8w* | A | C | 78.04 | 1.88 | 20.08 |
| 1m8x | A | C | 72.69 | 2.20 | 25.11 | 1m8y | A | C | 73.64 | 1.52 | 24.84 |
| 1mji* | AB | CD | 22.10 | 28.42 | 49.48 | 1mms | AB | CD | 17.31 | 31.50 | 51.19 |
| 1mzp* | A | B | 10.77 | 45.48 | 43.75 | 1n35* | A | BC | 18.55 | 36.63 | 44.81 |
| 1n77 | A | C | 23.84 | 29.20 | 46.96 | 1n78 | A | C | 23.63 | 28.89 | 47.48 |
| 1o0b | A | B | 35.45 | 27.32 | 37.23 | 1o0c | A | B | 35.54 | 26.49 | 37.97 |
| 1ooa* | A | C | 48.98 | 38.55 | 12.47 | 1q2r* | ABCD | EF | 41.08 | 27.85 | 31.07 |
| 1qa6 | A | C | 14.08 | 33.40 | 52.52 | 1qf6* | A | B | 42.07 | 23.01 | 34.92 |
| 1qrs | A | B | 35.58 | 24.73 | 39.68 | 1qrt | A | B | 35.55 | 25.23 | 39.22 |
| 1qru | A | B | 35.33 | 25.72 | 38.95 | 1qtq | A | B | 35.24 | 26.96 | 37.80 |
| 1qu2 | A | T | 19.58 | 31.86 | 48.55 | 1qu3* | A | T | 16.19 | 31.97 | 51.84 |
| 1r3e | A | C | 31.52 | 39.30 | 29.19 | 1rc7 | A | BCDE | 9.19 | 28.14 | 62.67 |
| 1rlg | A | C | 60.64 | 23.38 | 15.98 | 1rpu* | AB | CD | 12.85 | 40.06 | 47.09 |
| 1s03* | H | A | 21.40 | 30.75 | 47.84 | 1sds* | A | D | 68.16 | 13.21 | 18.62 |
| 1ser* | AB | T | 12.83 | 46.05 | 41.12 | 1si3 | A | B | 21.63 | 48.88 | 29.49 |

| | | | | | | | | | | | |
|---|---|---|---|---|---|---|---|---|---|---|---|
| 1sj3 | P | R | 61.80 | 16.38 | 21.82 | 1sj4 | P | R | 62.00 | 16.61 | 21.39 |
| 1sjf | A | B | 61.20 | 17.06 | 21.73 | 1tfw | ABCD | EFGHIJ | 23.76 | 35.88 | 40.36 |
| 1ttt | A | D | 19.23 | 28.69 | 52.07 | 1u0b* | B | A | 27.20 | 28.69 | 44.11 |
| 1urn | A | P | 65.56 | 11.92 | 22.51 | 1vbx | A | B | 61.82 | 16.12 | 22.06 |
| 1vby | A | B | 62.87 | 15.59 | 21.54 | 1vbz | A | B | 62.21 | 15.56 | 22.22 |
| 1vc0 | A | B | 62.20 | 16.63 | 21.16 | 1vc6 | A | B | 61.80 | 16.37 | 21.83 |
| 1wmq | A | C | 59.86 | 4.78 | 35.36 | 1wne | A | BC | 24.72 | 31.15 | 44.13 |
| 1wpu | A | C | 58.33 | 2.66 | 39.00 | 1wrq | A | C | 60.21 | 3.47 | 36.32 |
| 1wsu* | ABC | EFG | 15.00 | 50.00 | 35.00 | 1y39* | A | C | 13.21 | 30.17 | 56.61 |
| 1ytu* | A | CD | 33.23 | 34.29 | 32.47 | 1yty | AB | CD | 46.30 | 21.80 | 31.91 |
| 1yvp | A | CDG | 47.87 | 30.77 | 21.36 | 1yyo | AB | CDEF | 8.72 | 28.62 | 62.66 |
| 1yz9 | AB | CDEF | 10.18 | 22.51 | 67.31 | 1zbh* | AD | EF | 34.32 | 42.35 | 23.32 |
| 1ze2 | AB | CD | 26.82 | 37.34 | 35.84 | 1zh5 | AB | CD | 47.05 | 21.64 | 31.31 |
| 1zho | E | F | 6.80 | 46.59 | 46.61 | 1zjw | A | B | 35.11 | 27.12 | 37.77 |
| 1zl3* | A | B | 32.26 | 39.28 | 28.46 | 2a8v* | B | E | 70.63 | 12.26 | 17.10 |
| 2ab4* | A | B | 32.38 | 41.25 | 26.37 | 2ann | A | B | 58.23 | 9.99 | 31.78 |
| 2anr | A | B | 59.24 | 12.90 | 27.86 | 2asb | A | B | 57.56 | 10.03 | 32.41 |
| 2atw* | A | B | 60.23 | 10.63 | 29.14 | 2az0 | AB | CD | 6.81 | 42.11 | 51.08 |
| 2az2* | AB | CD | 7.22 | 41.77 | 51.01 | 2azx* | A | C | 36.28 | 27.20 | 36.52 |
| 2b3j | AB | EF | 51.60 | 19.85 | 28.55 | 2bgg | A | PQ | 33.02 | 32.01 | 34.97 |
| 2bh2* | A | C | 47.91 | 19.05 | 33.05 | 2bte* | A | B | 21.05 | 36.34 | 42.61 |
| 2bx2* | L | R | 34.51 | 41.10 | 24.40 | 2csx* | A | C | 24.53 | 22.09 | 53.38 |
| 2ct8 | A | C | 25.46 | 25.56 | 48.98 | 2cv0* | A | C | 23.49 | 29.55 | 46.96 |
| 2cv1 | A | C | 23.97 | 28.74 | 47.29 | 2cv2 | A | C | 23.64 | 28.02 | 48.34 |
| 2db3 | B | F | 12.65 | 46.67 | 40.68 | 2dlc | X | Y | 58.89 | 9.86 | 31.25 |
| 2dr2 | A | B | 43.19 | 23.04 | 33.77 | 2dr5 | A | B | 21.20 | 37.21 | 41.59 |
| 2dr7 | A | B | 19.23 | 39.09 | 41.68 | 2dr8 | A | B | 23.54 | 38.69 | 37.76 |
| 2dr9 | A | B | 24.03 | 34.65 | 41.32 | 2dra | A | B | 23.41 | 35.71 | 40.88 |
| 2drb | A | B | 25.38 | 33.21 | 41.41 | 2dvi | A | B | 23.82 | 35.12 | 41.05 |
| 2dxi | A | C | 23.60 | 29.76 | 46.64 | 2e9r | X | AB | 19.77 | 34.49 | 45.74 |
| 2e9t | A | BC | 10.04 | 40.35 | 49.61 | 2e9z | A | BC | 17.54 | 35.44 | 47.02 |
| 2ec0 | A | BC | 12.59 | 40.52 | 46.89 | 2ez6 | AB | CD | 7.97 | 28.67 | 63.36 |
| 2f8k | A | B | 39.43 | 44.88 | 15.69 | 2f8s | A | CD | 45.07 | 25.26 | 29.66 |
| 2fk6* | A | R | 27.62 | 33.92 | 38.46 | 2fmt* | A | C | 28.01 | 28.36 | 43.63 |
| 2g4b* | A | B | 60.89 | 12.00 | 27.11 | 2gic* | ABCDE | R | 33.04 | 40.62 | 26.34 |
| 2gjw | AB | EFH | 40.77 | 24.75 | 34.48 | 2gxb | AB | EF | 8.08 | 60.98 | 30.94 |
| 2hvy | ACD | E | 38.01 | 31.67 | 30.32 | 2hw8* | A | B | 6.97 | 42.53 | 50.50 |
| 2hyi | ABCD | F | 18.63 | 40.45 | 40.93 | 2i82* | AB | EF | 31.30 | 31.52 | 37.19 |
| 2i91* | A | CD | 32.25 | 36.98 | 30.77 | 2ix1 | A | B | 51.00 | 22.60 | 26.40 |
| 2j0s* | A | E | 16.91 | 43.17 | 39.92 | 2jlu* | A | C | 21.82 | 35.24 | 42.94 |
| 2jlv | A | C | 23.82 | 34.56 | 41.63 | 2jlw | A | C | 29.86 | 37.06 | 33.09 |
| 2jlx | A | C | 29.88 | 33.07 | 37.05 | 2jly | A | C | 31.81 | 31.61 | 36.58 |
| 2jlz | A | C | 29.87 | 33.51 | 36.63 | 2nue | AB | C | 6.57 | 34.97 | 58.46 |
| 2nuf | AB | CD | 6.82 | 30.16 | 63.02 | 2nug | AB | CEDF | 12.21 | 28.98 | 58.82 |

| | | | | | | | | | | | |
|---|---|---|---|---|---|---|---|---|---|---|---|
| 2nz4 | B | FQ | 54.34 | 21.42 | 24.23 | 2oih | A | B | 60.60 | 18.23 | 21.17 |
| 2oj3 | A | B | 61.53 | 17.65 | 20.82 | 2ozb* | AB | C | 49.83 | 34.06 | 16.11 |
| 2pjp | A | B | 52.60 | 38.94 | 8.46 | 2ply | AB | CE | 29.16 | 47.81 | 23.03 |
| 2po1 | AB | C | 34.64 | 34.78 | 30.58 | 2pxb | A | B | 37.82 | 13.57 | 48.61 |
| 2pxd | A | B | 36.65 | 14.54 | 48.82 | 2pxe | A | B | 38.61 | 12.87 | 48.52 |
| 2pxf | A | B | 37.17 | 13.28 | 49.56 | 2pxk | A | B | 36.19 | 15.61 | 48.20 |
| 2pxl | A | B | 36.66 | 14.07 | 49.27 | 2pxp | A | B | 37.83 | 13.04 | 49.13 |
| 2pxq | A | B | 37.01 | 13.38 | 49.62 | 2pxt | A | B | 38.68 | 13.98 | 47.34 |
| 2pxu | A | B | 37.53 | 13.31 | 49.15 | 2pxv | A | B | 35.71 | 17.54 | 46.75 |
| 2py9* | ABCD | EF | 62.12 | 9.17 | 28.70 | 2qux* | AB | C | 58.66 | 24.71 | 16.63 |
| 2r7r | A | X | 49.91 | 20.78 | 29.31 | 2r7t | A | X | 50.54 | 22.17 | 27.29 |
| 2r7v | A | X | 33.31 | 38.69 | 27.99 | 2r7w | A | X | 49.99 | 20.73 | 29.28 |
| 2r7x | A | X | 50.40 | 20.81 | 28.79 | 2r8s* | HL | R | 31.24 | 27.89 | 40.87 |
| 2rd2 | A | B | 35.14 | 26.99 | 37.88 | 2re8 | A | B | 34.91 | 26.78 | 38.31 |
| 2rfk | ABC | DEF | 27.45 | 30.74 | 41.81 | 2uwm | AB | CD | 28.95 | 46.79 | 24.26 |
| 2v3c* | AC | M | 21.76 | 39.35 | 38.89 | 2vnu | D | B | 42.53 | 28.57 | 28.90 |
| 2vod | A | C | 62.33 | 17.55 | 20.13 | 2von | A | C | 62.33 | 18.56 | 19.11 |
| 2vpl | A | B | 7.94 | 38.84 | 53.22 | 2xbm | AC | F | 43.90 | 46.90 | 9.20 |
| 2xd0 | A | G | 50.38 | 30.01 | 19.61 | 2xdb* | A | G | 43.56 | 35.81 | 20.63 |
| 2xli* | A | B | 13.00 | 45.00 | 42.00 | 2xlj | A | B | 36.66 | 50.05 | 13.29 |
| 2xlk | A | C | 37.89 | 47.28 | 14.83 | 2xs2* | A | B | 56.81 | 18.80 | 24.39 |
| 2xs7 | A | B | 56.04 | 18.81 | 25.15 | 2xzl | A | B | 37.70 | 31.10 | 31.20 |
| 2xzo* | A | D | 31.27 | 37.48 | 31.25 | 2y8w | A | B | 51.28 | 30.47 | 18.25 |
| 2y8y* | A | B | 40.53 | 43.74 | 15.72 | 2y9h | A | B | 31.05 | 49.04 | 19.91 |
| 2ykg | A | CD | 10.33 | 41.51 | 48.16 | 2zh1 | A | B | 19.75 | 39.38 | 40.88 |
| 2zh2 | A | B | 21.86 | 37.42 | 40.72 | 2zh3 | A | B | 22.70 | 36.21 | 41.09 |
| 2zh4 | A | B | 23.49 | 36.46 | 40.04 | 2zh5 | A | B | 20.98 | 36.81 | 42.20 |
| 2zh6 | A | B | 23.40 | 36.00 | 40.60 | 2zh7 | A | B | 20.22 | 38.81 | 40.98 |
| 2zh8 | A | B | 20.88 | 37.38 | 41.74 | 2zh9* | A | B | 19.11 | 38.90 | 41.99 |
| 2zha | A | B | 21.59 | 39.59 | 38.82 | 2zi0* | AB | CD | 31.06 | 62.86 | 6.08 |
| 2zko* | AB | CD | 8.43 | 31.26 | 60.30 | 2zm5 | A | C | 34.46 | 36.19 | 29.35 |
| 2zue* | A | B | 42.66 | 22.13 | 35.20 | 2zuf | A | B | 43.66 | 21.83 | 34.51 |
| 2zxu | A | C | 34.44 | 36.68 | 28.89 | 2zzm | A | B | 38.70 | 28.82 | 32.49 |
| 2zzn* | A | C | 33.41 | 34.29 | 32.30 | 3a6p | A | DE | 16.91 | 34.64 | 48.44 |
| 3adb | A | C | 24.69 | 35.44 | 39.87 | 3adc* | A | C | 38.69 | 35.98 | 25.33 |
| 3add | A | C | 24.27 | 35.13 | 40.60 | 3adl | A | BC | 12.08 | 34.83 | 53.08 |
| 3aev* | B | C | 59.02 | 14.72 | 26.26 | 3akz | A | E | 25.06 | 30.47 | 44.47 |
| 3am1 | A | B | 37.53 | 34.82 | 27.65 | 3amt* | A | B | 44.91 | 34.05 | 21.04 |
| 3avu | A | GT | 16.19 | 40.75 | 43.06 | 3avw* | A | GT | 13.27 | 36.24 | 50.49 |
| 3avx | A | GT | 16.37 | 36.05 | 47.58 | 3avy | A | GT | 20.80 | 32.99 | 46.21 |
| 3boy* | ABC | D | 62.63 | 5.44 | 31.93 | 3bsb | B | C | 68.91 | 5.44 | 25.66 |
| 3bsn | A | PT | 16.39 | 40.50 | 43.11 | 3bso | A | PT | 16.39 | 39.79 | 43.82 |
| 3bsx | A | C | 72.37 | 4.03 | 23.60 | 3bt7* | AB | CD | 29.33 | 30.73 | 39.94 |
| 3bx2* | A | C | 69.34 | 2.42 | 28.23 | 3bx3 | A | C | 73.56 | 2.04 | 24.41 |

| | | | | | | | | | | | |
|---|---|---|---|---|---|---|---|---|---|---|---|
| 3cul | AB | CD | 69.49 | 12.66 | 17.84 | 3cun | AB | CD | 67.71 | 13.35 | 18.94 |
| 3d2s | A | E | 85.10 | 4.17 | 10.73 | 3dd2* | H | B | 37.70 | 42.50 | 19.80 |
| 3dh3* | AB | EF | 28.00 | 35.91 | 36.09 | 3egz | A | B | 62.32 | 19.17 | 18.52 |
| 3eph* | A | E | 31.04 | 39.25 | 29.71 | 3eqt* | AB | CD | 23.59 | 38.11 | 38.30 |
| 3ex7 | ABCD | F | 28.48 | 35.45 | 36.08 | 3fht | A | C | 29.77 | 35.31 | 34.92 |
| 3foz* | B | D | 38.25 | 33.35 | 28.40 | 3ftf* | A | CD | 3.53 | 46.51 | 49.97 |
| 3g0h | A | E | 31.56 | 33.66 | 34.78 | 3g8t | B | FQ | 52.41 | 18.84 | 28.75 |
| 3g9c | B | FQ | 53.51 | 20.13 | 26.36 | 3h5x* | A | PT | 16.18 | 39.65 | 44.17 |
| 3h5y | A | PT | 15.67 | 40.50 | 43.84 | 3hax | ACD | EF | 30.09 | 35.74 | 34.17 |
| 3hhn | B | C | 62.27 | 17.24 | 20.49 | 3hjw | ABC | DE | 32.16 | 32.98 | 34.86 |
| 3i5x | A | B | 31.44 | 31.06 | 37.50 | 3i5y | A | B | 32.14 | 32.42 | 35.44 |
| 3i61 | A | B | 32.33 | 31.10 | 36.57 | 3i62 | A | B | 30.94 | 31.26 | 37.80 |
| 3iab* | AB | R | 58.17 | 23.39 | 18.44 | 3ice | ABCDEF | G | 22.40 | 35.17 | 42.42 |
| 3iev* | A | D | 61.22 | 10.57 | 28.21 | 3irw | P | R | 58.12 | 19.96 | 21.92 |
| 3k49* | A | B | 73.50 | 5.01 | 21.48 | 3k5q | A | B | 65.56 | 7.53 | 26.91 |
| 3k5y | A | B | 69.71 | 3.19 | 27.10 | 3k5z | A | B | 66.61 | 6.14 | 27.25 |
| 3k61 | A | B | 65.23 | 6.29 | 28.48 | 3k62 | A | B | 67.03 | 5.01 | 27.95 |
| 3k64 | A | B | 66.21 | 6.00 | 27.80 | 3kfu | ABCDEFGHIJ | KLMN | 37.96 | 29.95 | 32.09 |
| 3klv | A | BC | 20.44 | 33.71 | 45.85 | 3kms | A | BC | 17.70 | 35.23 | 47.07 |
| 3kna | A | BC | 20.71 | 33.86 | 45.44 | 3koa | A | BC | 12.45 | 41.45 | 46.09 |
| 3ks8 | AC | EF | 16.99 | 36.33 | 46.68 | 3l25 | ABDE | CF | 18.09 | 34.84 | 47.06 |
| 3l26 | AB | C | 21.13 | 41.31 | 37.56 | 3l3c | B | FQ | 52.70 | 21.61 | 25.69 |
| 3lqx | A | B | 37.60 | 14.25 | 48.15 | 3lrn | AB | CD | 19.62 | 50.70 | 29.68 |
| 3lrr | A | C | 20.33 | 46.20 | 33.47 | 3lwo | ABC | DE | 33.60 | 30.76 | 35.64 |
| 3lwp | ABC | DE | 32.53 | 32.87 | 34.60 | 3lwq* | ABC | DE | 33.47 | 31.22 | 35.31 |
| 3lwr | ABC | DE | 33.69 | 31.07 | 35.23 | 3lwv | ABC | DE | 33.90 | 30.99 | 35.11 |
| 3m7n | ABCDEFGHI | XYZ | 32.15 | 30.90 | 36.95 | 3m85 | ABCDEFGHI | XYZ | 29.84 | 36.92 | 33.24 |
| 3mdg* | A | C | 62.62 | 13.08 | 24.30 | 3mdi | AB | C | 57.41 | 14.70 | 27.89 |
| 3mj0 | A | B | 34.50 | 35.70 | 29.80 | 3moj* | B | A | 52.00 | 28.28 | 19.71 |
| 3mqk | A | DE | 35.18 | 31.65 | 33.17 | 3mum | P | R | 63.29 | 15.69 | 21.02 |
| 3mur | P | R | 58.32 | 19.68 | 22.00 | 3mut | P | R | 62.56 | 18.38 | 19.06 |
| 3mxh | P | R | 60.72 | 17.17 | 22.12 | 3ncu | AB | CD | 25.72 | 46.71 | 27.56 |
| 3ndb | AB | M | 27.55 | 35.46 | 37.00 | 3nl0* | A | BC | 14.34 | 37.62 | 48.04 |
| 3nmr | A | B | 64.76 | 13.72 | 21.52 | 3nmu | AGF | DI | 45.35 | 28.59 | 26.06 |
| 3nna | A | B | 66.25 | 12.19 | 21.56 | 3nnc* | A | B | 70.12 | 10.36 | 19.51 |
| 3nnh | AC | E | 64.38 | 10.50 | 25.12 | 3nvi* | AB | E | 43.73 | 29.98 | 26.29 |
| 3o3i* | X | A | 34.70 | 24.67 | 40.63 | 3o6e | X | A | 38.13 | 17.17 | 44.70 |
| 3o7v | X | A | 39.60 | 15.39 | 45.01 | 3o8c* | A | C | 33.18 | 36.82 | 29.99 |
| 3o8r | A | BC | 33.78 | 34.28 | 31.94 | 3og8 | AB | CD | 21.59 | 48.81 | 29.61 |
| 3oij | AB | CD | 50.09 | 16.31 | 33.60 | 3oin | AB | C | 48.59 | 16.76 | 34.65 |
| 3ol6 | A | BC | 19.66 | 34.75 | 45.59 | 3ol7 | A | BC | 20.32 | 34.02 | 45.66 |
| 3ol8 | A | BC | 20.23 | 33.85 | 45.92 | 3ol9 | A | B | 26.38 | 29.94 | 43.68 |
| 3ola | A | BC | 19.05 | 34.74 | 46.21 | 3olb | A | BC | 19.63 | 34.65 | 45.71 |
| 3ouy | A | C | 26.35 | 31.33 | 42.32 | 3ov7 | AB | CD | 24.42 | 33.02 | 42.56 |

| | | | | | | | | | | | |
|---|---|---|---|---|---|---|---|---|---|---|---|
| 3ova | A | C | 23.72 | 35.91 | 40.36 | 3ovb | A | C | 29.57 | 29.09 | 41.34 |
| 3ovs | A | C | 25.74 | 32.75 | 41.51 | 3pew | A | B | 26.75 | 37.80 | 35.45 |
| 3pey | A | B | 29.92 | 36.41 | 33.67 | 3pf4 | B | R | 40.01 | 4.68 | 55.30 |
| 3q0n | A | C | 72.26 | 2.29 | 25.45 | 3q0o | A | C | 74.39 | 1.21 | 24.40 |
| 3q0q | A | B | 71.28 | 5.75 | 22.97 | 3q0r | A | B | 70.80 | 5.69 | 23.51 |
| 3q0s | A | B | 71.68 | 1.69 | 26.63 | 3qg9 | A | B | 71.05 | 4.63 | 24.32 |
| 3qgb | A | B | 71.42 | 2.18 | 26.40 | 3qgc | A | B | 70.19 | 2.96 | 26.86 |
| 3qjj* | A | Q | 71.29 | 11.81 | 16.89 | 3qjl | AB | XR | 64.04 | 16.79 | 19.17 |
| 3qrp | A | BC | 42.11 | 38.14 | 19.76 | 3r2c* | A | R | 74.36 | 5.74 | 19.92 |
| 3r2d* | ABJK | RS | 6.64 | 45.12 | 48.23 | 3r9w* | A | B | 60.11 | 16.03 | 23.85 |
| 3r9x | AB | C | 49.97 | 23.99 | 26.04 | 3rc8* | A | E | 25.71 | 44.70 | 29.59 |
| 3rer | ABCDEF | K | 49.73 | 17.70 | 32.57 | 3rw6* | AB | FH | 30.65 | 32.84 | 36.51 |
| 3siu | AB | C | 53.05 | 29.49 | 17.46 | 3sn2 | A | B | 37.04 | 28.12 | 34.84 |
| 3snp* | AB | CD | 37.93 | 28.81 | 33.25 | 3sqw | A | B | 16.39 | 44.71 | 38.90 |
| 3t5n* | A | C | 37.36 | 37.37 | 25.27 | 3t5q | A | C | 42.22 | 35.61 | 22.17 |
| 3tmi* | A | BC | 7.95 | 43.49 | 48.56 | 3trz | AB | UV | 71.79 | 7.85 | 20.36 |
| 3ts0 | AB | UV | 70.14 | 10.86 | 19.00 | 3ts2* | AB | UV | 75.11 | 5.25 | 19.64 |
| 3u4m | A | B | 10.71 | 40.02 | 49.27 | 3u56 | A | B | 8.72 | 44.05 | 47.22 |
| 3ucu | P | AR | 57.85 | 19.42 | 22.73 | 3ucz | P | R | 60.83 | 18.40 | 20.77 |
| 3ud4 | P | R | 59.91 | 19.15 | 20.94 | 3umy | A | B | 11.67 | 43.44 | 44.89 |
| 3v6y | A | B | 70.09 | 7.02 | 22.90 | 3v71* | A | B | 62.42 | 9.47 | 28.11 |
| 3v74* | A | B | 72.59 | 4.14 | 23.27 | 3v7e* | A | C | 67.69 | 20.66 | 11.65 |
| 3vjr* | A | B | 13.44 | 36.26 | 50.30 | 3vnv | A | GT | 14.99 | 33.05 | 51.96 |
| 3vyy* | AB | CDEF | 7.27 | 34.75 | 57.98 | 3wbm* | ABCD | XY | 8.68 | 55.34 | 35.98 |
| 3zc0* | AB | M | 4.44 | 66.17 | 29.39 | 3zd6 | A | CD | 11.69 | 46.72 | 41.59 |
| 3zd7 | A | CD | 11.21 | 36.94 | 51.85 | 3zgz | A | B | 30.46 | 30.46 | 39.08 |
| 3zjt | A | B | 26.21 | 28.65 | 45.15 | 3zju | A | B | 28.71 | 27.55 | 43.74 |
| 3zjv | A | B | 32.28 | 25.71 | 42.01 | 4al5 | A | B | 39.56 | 42.09 | 18.35 |
| 4al6 | A | B | 40.81 | 43.12 | 16.07 | 4am3 | ABC | E | 57.42 | 15.38 | 27.19 |
| 4aq7 | A | B | 30.06 | 30.47 | 39.47 | 4arc* | A | B | 30.85 | 31.78 | 37.37 |
| 4ari | A | B | 26.67 | 31.68 | 41.66 | 4as1 | A | B | 31.80 | 27.50 | 40.70 |
| 4ato* | A | G | 46.79 | 32.40 | 20.81 | 4ay2 | A | C | 9.98 | 44.86 | 45.16 |
| 4b3g | A | G | 42.45 | 29.09 | 28.47 | 4bpb | A | CD | 10.36 | 43.21 | 46.43 |
| 4bw0 | B | A | 52.86 | 30.99 | 16.14 | 4c4w | A | D | 62.00 | 20.20 | 17.80 |
| 4c7o* | BD | E | 31.61 | 22.33 | 46.06 | 4c8z | A | C | 40.48 | 32.68 | 26.84 |
| 4c9d | A | C | 48.12 | 26.56 | 25.31 | 4e78* | A | PT | 11.44 | 35.63 | 52.92 |
| 4e7a | A | PT | 13.67 | 38.72 | 47.62 | 4ed5* | A | D | 62.37 | 14.32 | 23.31 |
| 4erd* | AB | CD | 58.40 | 20.83 | 20.77 | 4f3t* | A | R | 30.86 | 34.35 | 34.78 |
| 4fvu* | A | BC | 19.67 | 34.31 | 46.01 | 4gcw | A | B | 25.97 | 35.83 | 38.19 |
| 4gha* | ACEG | IJ | 1.70 | 34.98 | 63.32 | 4ghl | CD | EF | 1.83 | 35.61 | 62.56 |
| 4h5p* | AB | E | 48.03 | 26.40 | 25.57 | 4ifd | ABCDEFGHIJK | R | 29.00 | 29.95 | 41.05 |
| 4ig8* | A | BC | 7.05 | 35.42 | 57.52 | 4ill* | AB | CR | 49.01 | 27.07 | 23.92 |
| 4iqx | A | BC | 18.22 | 35.03 | 46.76 | 4j1g | ABCD | E | 42.92 | 26.20 | 30.88 |
| 4j39 | A | B | 16.76 | 38.39 | 44.86 | 4j5v | A | B | 17.60 | 37.34 | 45.06 |

| | | | | | | | | | | | |
|---|---|---|---|---|---|---|---|---|---|---|---|
| 4jgn | A | E | 19.97 | 40.27 | 39.76 | 4jk0 | D | B | 22.34 | 36.93 | 40.73 |
| 4jng* | ABCD | L | 42.87 | 24.36 | 32.76 | 4jnx | AD | BG | 28.64 | 26.85 | 44.52 |
| 4jvy* | A | D | 56.82 | 10.91 | 32.27 | 4jxx | A | B | 35.97 | 26.63 | 37.40 |
| 4jxz | A | B | 34.68 | 27.33 | 37.99 | 4jyz | A | B | 33.31 | 27.67 | 39.01 |
| 4k4s | AE | BCFG | 18.45 | 34.99 | 46.56 | 4k4t* | A | BC | 18.81 | 34.02 | 47.16 |
| 4k4u | A | BC | 18.68 | 32.49 | 48.83 | 4k4v | A | BC | 17.90 | 35.70 | 46.40 |
| 4k4w | A | BC | 20.07 | 33.45 | 46.48 | 4k4x | A | BC | 20.30 | 32.48 | 47.23 |
| 4k4y | A | BC | 19.15 | 34.70 | 46.15 | 4k4z | A | BC | 20.13 | 34.29 | 45.59 |
| 4k50* | A | BC | 20.21 | 32.07 | 47.72 | 4knq | A | B | 18.70 | 39.63 | 41.67 |
| 4kq0 | AD | BE | 14.69 | 37.12 | 48.19 | 4kr6* | AB | CD | 34.21 | 23.88 | 41.91 |
| 4kre | A | R | 29.91 | 34.26 | 35.83 | 4krf | A | R | 31.72 | 35.40 | 32.88 |
| 4kxt | A | B | 28.15 | 36.66 | 35.19 | 4kzd | HL | R | 54.08 | 26.01 | 19.91 |
| 4kze | HL | R | 54.48 | 26.24 | 19.28 | 4l8h | AB | R | 35.53 | 39.62 | 24.85 |
| 4l8r | E | D | 35.93 | 35.59 | 28.49 | 4lg2 | B | EF | 32.94 | 41.34 | 25.72 |
| 4lgt* | A | E | 40.74 | 29.78 | 29.48 | 4lmz | A | B | 56.26 | 16.30 | 27.44 |
| 4m2z | AB | CD | 8.76 | 28.91 | 62.33 | 4m30 | AB | CD | 7.64 | 28.66 | 63.70 |
| 4m59* | AB | CD | 43.93 | 18.34 | 37.72 | 4m7a | ABCDEFG | P | 67.92 | 10.62 | 21.46 |
| 4m7d | ABCDEFG | P | 68.48 | 9.91 | 21.60 | 4mdx* | AB | C | 63.76 | 14.47 | 21.77 |
| 4n0t* | A | B | 54.87 | 17.46 | 27.67 | 4n2q | A | B | 40.84 | 34.94 | 24.22 |
| 4ngb | A | B | 39.53 | 38.53 | 21.94 | 4ngc* | A | B | 36.47 | 39.98 | 23.54 |
| 4ngd | A | B | 38.75 | 38.65 | 22.60 | 4ngg | A | B | 34.44 | 29.47 | 36.09 |
| 4nh3 | A | B | 41.76 | 36.03 | 22.21 | 4nh5 | A | B | 44.42 | 30.88 | 24.69 |
| 4nh6 | A | B | 43.53 | 33.94 | 22.53 | 4o26 | AB | EF | 34.60 | 28.24 | 37.15 |
| 4o8j | A | E | 38.76 | 35.16 | 26.08 | 4oe1 | AB | DC | 43.93 | 18.42 | 37.65 |
| 4ola | A | B | 25.98 | 39.20 | 34.82 | 4olb | A | B | 26.26 | 38.65 | 35.09 |
| 4oog* | C | D | 23.01 | 30.55 | 46.44 | | | | | | |

* The 137 non-redundant complexes are marked with an asterisk(*)

**Table S2.** Detailed information of 137 protein-RNA complexes including the final data and all of properties.

Chain id: Chain ids in the PDB entry.

Res(Å): Resolution of the X-ray structure.

RNA type: We only considered the four common types of RNA, ssRNA, dsRNA, tRNA and rRNA, respectively, mRNA was classified as ssRNA in our study.

PISA(kcal/Mol): The solvation free energy gain upon formation of the interface.

B(Å2): Interface area.

Surface area B ratio (%): The ratio of the interface area to the rest of the surface area.

N_atoms: Number of interface atoms.

N_residues/N_nucleotides: Number of interface amino acid residues and interface nucleotides.

N_hb: Number of H-bonds between amino acids and nucleotides at the protein-RNA interface.

P_e(%): Percent-overlap between the largest electrostatic positive patches on protein surfaces and the binding interface.

EEL(kcal/mol): Electrostatic energy.

N_stacking: Number of stacking interactions at protein-RNA interface.

N_vdw: Number of van der waals contacts.

N_hp: Number of hydrophobic interactions.

P_hp(%): Percentage of hydrophobic interface in total binding interface.

| | Chain id | | Res(Å) | RNA type | PISA | B(Å2) | | | Surface area B ratio (%) | | | N_atoms | | | N_residues / N_nucleotides | | N_hb | P_e(%) | EEL | N_stacking | N_vdw | N_hp | P_hp(%) |
|---|---|---|---|---|---|---|---|---|---|---|---|---|---|---|---|---|---|---|---|---|---|---|---|
| | Pro | RNA | | | | All | Pro | RNA | All | Pro | RNA | All | Pro | RNA | Pro | RNA | | | | | | | |
| High | | | | | | | | | | | | | | | | | | | | | | | |
| 1B7F | A | P | 2.6 | Miscellaneous | -21.7 | 2937 | 1339.2 | 1597.8 | 28.1 | 13.4 | 46.7 | 320 | 173 | 147 | 48 | 10 | 24 | 96 | -2112.2 | 5 | 189 | 379 | 79 |
| 1E7K | A | C | 2.9 | sRNA | -8.3 | 1299.9 | 620.5 | 679.4 | 14.5 | 8.7 | 21.7 | 148 | 68 | 80 | 19 | 9 | 15 | 74 | -2061.8 | 0 | 88 | 99 | 74 |
| 1EC6 | A | D | 2.4 | ssRNA | -8 | 1854 | 905.9 | 948 | 22.4 | 15.4 | 22.3 | 205 | 97 | 108 | 30 | 13 | 13 | 83 | -2247.3 | 1 | 95 | 166 | 67 |
| 1GTF | LMNOPQRST | W | 1.75 | mRNA | -20.2 | 7913 | 3590.6 | 4322.4 | 25.5 | 14 | 32.6 | 901 | 462 | 439 | 162 | 39 | 71 | 38 | 851.8 | 9 | 384 | 949 | 51 |
| 1KQ2 | ABHIKM | R | 2.71 | rRNA | -33.6 | 3025.6 | 1353.6 | 1671.9 | 17.8 | 7.6 | 76.2 | 320 | 190 | 130 | 44 | 7 | 22 | 39 | -185.0 | 5 | 182 | 414 | 84 |
| 1M8V | ABCDEFGHI | OPQRSTU | 2.6 | Miscellaneous | -10 | 6572.7 | 2984 | 3588.7 | 19.5 | 10 | 34.8 | 734 | 379 | 355 | 111 | 27 | 87 | 18 | -3751.3 | 14 | 208 | 475 | 28 |
| 1M8W | A | C | 2.2 | mRNA | -2.7 | 2109.8 | 946.5 | 1163.3 | 12.3 | 5.7 | 43.5 | 217 | 113 | 104 | 38 | 7 | 11 | 0 | -402.2 | 10 | 149 | 255 | 71 |
| 1SDS | A | D | 1.8 | mRNA | -9 | 851.7 | 369.4 | 482.4 | 10 | 6.4 | 13.3 | 89 | 40 | 49 | 16 | 4 | 9 | 25 | -120.8 | 0 | 51 | 91 | 94 |
| 2A8V | B | E | 2.4 | ssRNA | -2.2 | 719.2 | 281.1 | 438.1 | 8.6 | 3.9 | 23.1 | 88 | 52 | 36 | 16 | 4 | 8 | 25 | 64.7 | 2 | 32 | 117 | 63 |
| 2ATW | A | B | 2.25 | dsRNA | -5.9 | 2574.9 | 1192.4 | 1382.5 | 20 | 9.8 | 42 | 307 | 152 | 155 | 45 | 11 | 16 | 0 | -1020.0 | 0 | 145 | 325 | 91 |
| 2G4B | A | B | 2.5 | rRNA | -8.6 | 1161.4 | 542.4 | 619 | 10.9 | 5.6 | 28.9 | 134 | 70 | 64 | 20 | 5 | 9 | 30 | -197.6 | 3 | 46 | 127 | 55 |
| 2PY9 | ABCD | EF | 2.56 | microRNA | -7.6 | 3345.6 | 1606.7 | 1739 | 17.8 | 9.8 | 30.7 | 372 | 179 | 193 | 60 | 19 | 10 | 63 | -3196.6 | - | - | - | - |
| 2QUX | AB | C | 2.44 | Miscellaneous | 0.1 | 1753.6 | 800.8 | 952.7 | 11.2 | 6.5 | 18.9 | 185 | 89 | 96 | 35 | 11 | 21 | 34 | -3122.4 | 3 | 105 | 125 | 69 |
| 2XS2 | A | B | 1.35 | mRNA | -9.8 | 1298.2 | 606.3 | 691.9 | 19.7 | 9.9 | 38.7 | 159 | 81 | 78 | 22 | 6 | 7 | 57 | -758.5 | 3 | 70 | 181 | 81 |
| 3AEV | B | C | 2.8 | mRNA | -9 | 2416 | 1131.3 | 1284.7 | 21.7 | 10.8 | 41.7 | 260 | 133 | 127 | 40 | 11 | 19 | 97 | -2285.7 | 1 | 131 | 228 | 75 |
| 3BOY | ABC | D | 1.7 | Miscellaneous | -31.9 | 4682.6 | 2082.1 | 2600.5 | 20.3 | 9.6 | 43.9 | 587 | 312 | 275 | 92 | 22 | 29 | 7 | - | 0 | 305 | 638 | 78 |
| 3BX2 | A | C | 2.84 | mRNA | -5 | 2466.8 | 1097.3 | 1369.5 | 15.7 | 7.2 | 48.5 | 249 | 129 | 120 | 42 | 9 | 18 | 0 | 30.7 | 10 | 170 | 316 | 71 |
| 3IAB | AB | R | 2.7 | snRNA | -20.8 | 5064.9 | 2387.7 | 2677.2 | 26.1 | 16.3 | 27.2 | 534 | 266 | 268 | 79 | 29 | 30 | 80 | -12294.9 | 0 | 50 | 113 | 35 |
| 3IEV | A | D | 1.9 | ssRNA | -1.4 | 2272.6 | 1013.3 | 1259.4 | 14.7 | 6.9 | 42 | 266 | 139 | 127 | 38 | 9 | 23 | 68 | -1670.3 | 2 | 140 | 330 | 82 |
| 3K49 | A | B | 2.5 | ssRNA | 3.1 | 3080.8 | 1380.1 | 1700.7 | 17.1 | 7.7 | 52 | 301 | 155 | 146 | 48 | 10 | 25 | 6 | 40.3 | 12 | 226 | 397 | 88 |
| 3MDG | A | C | 2.22 | mRNA | -6.8 | 1068.9 | 503.2 | 565.7 | 9.2 | 4.6 | 33.7 | 128 | 67 | 61 | 23 | 6 | 6 | 56 | -232.0 | 3 | 52 | 138 | 74 |
| 3NNC | A | B | 2.2 | ssRNA | -10.3 | 1145.8 | 517.8 | 628 | 10 | 4.9 | 32.1 | 113 | 60 | 53 | 22 | 5 | 4 | 59 | -475.3 | 2 | 40 | 156 | 77 |
| 3QJJ | A | Q | 2.8 | rRNA | -4.1 | 3190.7 | 1489.5 | 1701.2 | 25.5 | 12.1 | 50.6 | 348 | 179 | 169 | 50 | 12 | 26 | 88 | -2163.1 | 6 | 180 | 340 | 70 |
| 3R2C | A | R | 1.9 | mRNA | -8.7 | 2209.1 | 1004.1 | 1205 | 27.9 | 13.1 | 48.6 | 249 | 130 | 119 | 36 | 9 | 7 | 36 | -1285.9 | 4 | 948 | 321 | 81 |
| 3R9W | A | B | 2.05 | mRNA | -8.8 | 2717.1 | 1236 | 1481 | 13.7 | 8.6 | 18.3 | 306 | 152 | 154 | 44 | 15 | 23 | 75 | -3861.7 | 2 | 141 | 324 | 82 |
| 3TS2 | AB | UV | 2.01 | mRNA | -14.6 | 6881.8 | 3266.4 | 3615.4 | 32.1 | 19.4 | 31.3 | 764 | 372 | 392 | 90 | 37 | 50 | 81 | -9012.5 | 22 | 375 | 982 | 80 |
| 3V71 | A | B | 2.9 | rRNA | -10.8 | 1951.9 | 875.6 | 1076.3 | 10.9 | 4.9 | 50.9 | 202 | 106 | 96 | 30 | 7 | 17 | 75 | -317.7 | 6 | 142 | 249 | 87 |
| 3V74 | A | B | 2.3 | mRNA | -3.3 | 3038.1 | 1366.2 | 1672 | 15.7 | 7.2 | 49.8 | 312 | 161 | 151 | 47 | 11 | 24 | 17 | -772.3 | 12 | 192 | 386 | 87 |
| 3V7E | A | C | 2.8 | mRNA | -10 | 873.8 | 404.7 | 469.1 | 3.6 | 9.8 | 2.3 | 98 | 38 | 60 | 16 | 6 | 4 | 0 | -4086.2 | 0 | 37 | 82 | 88 |
| 4ED5 | A | D | 2 | Miscellaneous | -17.9 | 2516.6 | 1181.8 | 1334.8 | 25.6 | 11.9 | 55.4 | 272 | 147 | 125 | 43 | 10 | 23 | 91 | -1579.7 | 4 | 152 | 314 | 81 |
| 4ERD | AB | CD | 2.59 | mRNA | -10 | 3279.6 | 1589.8 | 1689.8 | 15.8 | 10.1 | 20.1 | 373 | 180 | 193 | 45 | 18 | 9 | 69 | -6427.5 | 0 | 13 | 16 | 6 |
| 4JVY | A | D | 2.85 | Miscellaneous | -3.2 | 1665.1 | 806.8 | 858.4 | 13.4 | 6.6 | 45.5 | 180 | 86 | 94 | 28 | 7 | 8 | 89 | -597.1 | 1 | 109 | 171 | 82 |
| 4MDX | AB | C | 1.5 | Miscellaneous | -8.7 | 2126.7 | 958.2 | 1168.5 | 18.9 | 8.9 | 44.7 | 207 | 125 | 82 | 41 | 7 | 14 | 0 | 32.1 | 1 | 120 | 248 | 96 |
| Medium | | | | | | | | | | | | - | | | | | | | | | | | |
| 1ASZ | A | R | 3 | tRNA | -31.9 | 3865.6 | 1837.4 | 2028.2 | 11.7 | 7.6 | 15.8 | 443 | 221 | 222 | 71 | 29 | 23 | 2 | 2042.6 | 0 | 100 | 113 | 28 |
| 1EFW | A | C | 3 | tRNA | -12.8 | 2044.7 | 1005 | 1039.7 | 5.2 | 3.4 | 8.8 | 237 | 119 | 118 | 39 | 19 | 16 | 43 | 1224.1 | 0 | 19 | 12 | 5 |
| 1F7U | A | B | 2.2 | tRNA | -30.6 | 5135.4 | 2386.2 | 2749.2 | 14.1 | 8.5 | 20.4 | 562 | 303 | 259 | 90 | 35 | 33 | 23 | -636.0 | 0 | 77 | 119 | 19 |
| 1GAX | A | C | 2.9 | tRNA | -27.2 | 5171.9 | 2501.7 | 2670.3 | 11.4 | 6.6 | 20.6 | 605 | 308 | 297 | 100 | 40 | 24 | 1 | 5384.4 | 1 | 316 | 376 | 53 |
| 1GTR | A | B | 2.5 | dsRNA | -31 | 5318.4 | 2513.5 | 2804.9 | 16.8 | 10.5 | 21.5 | 580 | 288 | 292 | 83 | 34 | 52 | 37 | 3442.9 | 0 | 362 | 391 | 70 |

| | | | | | | | | | | | | | | | | | | | | | | | |
|---|---|---|---|---|---|---|---|---|---|---|---|---|---|---|---|---|---|---|---|---|---|---|---|
| 1H3E | A | B | 2.9 | tRNA | -22.8 | 2223.8 | 1087.9 | 1135.9 | 7.1 | 5.5 | 8.4 | 263 | 136 | 127 | 43 | 23 | 0 | 5 | - | 0 | 9 | 16 | 7 |
| 1IL2 | A | C | 2.6 | Miscellaneous | -28 | 4085.8 | 1983.7 | 2102.2 | 11 | 6.9 | 16.6 | 449 | 228 | 221 | 72 | 27 | 40 | 1 | 4097.4 | 1 | 163 | 105 | 29 |
| 1OOA | A | C | 2.45 | tRNA | -17.1 | 1909.3 | 996.5 | 912.8 | 9.3 | 5.9 | 16.8 | 217 | 98 | 119 | 28 | 19 | 20 | 54 | -2651.5 | 3 | 78 | 108 | 57 |
| 1Q2R | ABCD | EF | 2.9 | vRNA | -18.5 | 6034.4 | 2841.3 | 3193.1 | 11 | 5.4 | 39.8 | 626 | 325 | 301 | 103 | 32 | 30 | 60 | -7793.2 | 0 | 430 | 472 | 61 |
| 1QF6 | A | B | 2.9 | snRNA | -29.3 | 4228.5 | 2079 | 2149 | 10.8 | 6.8 | 16.5 | 459 | 223 | 236 | 70 | 27 | 28 | 14 | 5924.2 | 2 | 106 | 156 | 37 |
| 1U0B | B | A | 2.3 | Miscellaneous | -27 | 4557.2 | 2150.9 | 2406.3 | 16.1 | 10.6 | 19.2 | 526 | 276 | 250 | 85 | 32 | 25 | 15 | - | 2 | 254 | 339 | 66 |
| 1YTU | A | CD | 2.5 | mRNA | -13.1 | 1851.6 | 800.2 | 1051.4 | 9.4 | 4.1 | 47.2 | 213 | 120 | 93 | 32 | 7 | 14 | 97 | -1174.4 | 0 | 56 | 29 | 16 |
| 1ZBH | AD | EF | 3 | crRNA | -32.7 | 3470.7 | 1744.5 | 1726.2 | 10.7 | 5.9 | 27.2 | 383 | 185 | 198 | 53 | 24 | 16 | 43 | -5873.2 | 2 | 211 | 215 | 57 |
| 1ZL3 | A | B | 2.8 | dsRNA | -17.9 | 2547.9 | 1207.3 | 1340.6 | 15.2 | 8 | 31.6 | 279 | 141 | 138 | 44 | 12 | 22 | 89 | -850.0 | 1 | 134 | 128 | 55 |
| 2AB4 | A | B | 2.4 | tRNA | -18 | 2557.2 | 1236.1 | 1321.1 | 15.4 | 8.1 | 34.4 | 289 | 149 | 140 | 44 | 12 | 18 | 91 | -3446.7 | 1 | 134 | 150 | 50 |
| 2AZX | A | C | 2.8 | tRNA | -10.2 | 1363.2 | 648.2 | 715 | 4.8 | 3.7 | 5.9 | 156 | 82 | 74 | 23 | 12 | 12 | 4 | 2043.4 | - | - | - | - |
| 2BH2 | A | C | 2.15 | tRNA | -14.5 | 4042.5 | 1978.9 | 2063.6 | 18.9 | 10.3 | 33.4 | 436 | 224 | 212 | 70 | 24 | 36 | 83 | -2537.9 | 4 | 206 | 288 | 50 |
| 2BX2 | L | R | 2.85 | tRNA | -10.3 | 912.2 | 419 | 493.2 | 3.1 | 1.6 | 13.4 | 94 | 50 | 44 | 20 | 4 | 5 | 100 | - | 0 | 67 | 67 | 50 |
| 2CSX | A | C | 2.7 | tRNA | -12.7 | 2182.3 | 1040.6 | 1141.8 | 7 | 5 | 8.9 | 260 | 129 | 131 | 40 | 22 | 15 | 20 | -3755.6 | 3 | 152 | 206 | 53 |
| 2CV0 | A | C | 2.4 | Miscellaneous | -34.2 | 4517 | 2117.3 | 2399.8 | 15.1 | 9.8 | 18.8 | 506 | 265 | 241 | 88 | 33 | 34 | 36 | -747.7 | 2 | 303 | 329 | 63 |
| 2FK6 | A | R | 2.9 | tRNA | -9.5 | 1531.1 | 750.6 | 780.5 | 6.9 | 5.2 | 8.5 | 194 | 91 | 103 | 23 | 17 | 9 | 35 | 1312.6 | 0 | 90 | 122 | 70 |
| 2FMT | A | C | 2.8 | rRNA | -19.2 | 2940.7 | 1498.5 | 1442.2 | 11.5 | 9.8 | 11 | 319 | 151 | 168 | 43 | 25 | 24 | 26 | -2010.8 | 1 | 122 | 154 | 56 |
| 2GIC | ABCDE | R | 2.92 | piRNA | -76.8 | 10344.6 | 5098.9 | 5245.7 | 10.6 | 5.2 | 48.5 | 1150 | 579 | 571 | 185 | 45 | 61 | 79 | -6111.6 | 5 | 731 | 668 | 45 |
| 2I82 | AB | EF | 2.05 | Miscellaneous | -26.9 | 5812 | 2852.2 | 2959.8 | 23.2 | 12.6 | 36.1 | 615 | 329 | 286 | 86 | 26 | 26 | 93 | -6584.1 | - | - | - | - |
| 2I91 | A | CD | 2.65 | Miscellaneous | -29.4 | 3893.6 | 1856.5 | 2037.1 | 16.4 | 8 | 44.6 | 432 | 221 | 211 | 65 | 17 | 31 | 94 | -3919.1 | 2 | 297 | 251 | 63 |
| 2OZB | AB | C | 2.6 | mRNA | -26.5 | 2569 | 1261.3 | 1307.8 | 11 | 6.4 | 21.1 | 272 | 126 | 146 | 42 | 17 | 20 | 71 | -3058.0 | 1 | 148 | 200 | 63 |
| 2R8S | HL | R | 1.95 | mRNA | -25.2 | 2509.9 | 1193.6 | 1316.2 | 6 | 6.3 | 5.1 | 271 | 128 | 133 | 42 | 27 | 24 | 17 | -8204.8 | 0 | 162 | 204 | 74 |
| 2XDB | A | G | 2.55 | ssRNA | -20 | 1882.8 | 875 | 1007.8 | 13.6 | 9.9 | 14.8 | 210 | 105 | 105 | 34 | 11 | 12 | 79 | -3026.9 | 1 | 88 | 158 | 53 |
| 2XZO | A | D | 2.4 | Miscellaneous | -30.7 | 2005.8 | 937.1 | 1068.7 | 7.4 | 3.4 | 65.6 | 209 | 114 | 95 | 42 | 7 | 18 | 83 | - | 0 | 127 | 163 | 62 |
| 2Y8Y | A | B | 1.44 | Miscellaneous | -19.9 | 2167.1 | 1065.7 | 1101.4 | 16.7 | 9.7 | 26.7 | 234 | 118 | 116 | 35 | 16 | 17 | 100 | -4021.5 | 0 | 32 | 45 | 23 |
| 2ZI0 | AB | CD | 2.82 | microRNA | -44.9 | 4573.3 | 2340.1 | 2233.2 | 31 | 19.4 | 30.7 | 470 | 208 | 262 | 54 | 39 | 43 | 95 | - | 5 | 321 | 209 | 54 |
| 2ZUE | A | B | 2 | ssRNA | -21.5 | 4591.6 | 2226 | 2365.6 | 12.2 | 7.6 | 18.4 | 509 | 249 | 260 | 76 | 39 | 25 | 6 | 2992.4 | 0 | 276 | 364 | 61 |
| 2ZZN | A | C | 2.95 | Miscellaneous | -27.3 | 4361.2 | 2162.4 | 2198.8 | 16.5 | 11.8 | 17.7 | 478 | 238 | 240 | 71 | 35 | 31 | 92 | -14109.7 | 0 | 271 | 310 | 55 |
| 3ADC | A | C | 2.9 | Miscellaneous | -26.6 | 3386.9 | 1733.5 | 1653.4 | 12 | 10.4 | 11 | 371 | 176 | 195 | 52 | 32 | 24 | 46 | -10474.6 | - | - | - | - |
| 3AMT | A | B | 2.9 | tRNA | -17.2 | 3818.9 | 1826.2 | 1992.7 | 12.4 | 8.7 | 14.5 | 410 | 219 | 191 | 64 | 26 | 29 | 28 | - | - | - | - | - |
| 3BT7 | AB | CD | 2.43 | rRNA | -32.3 | 3593.8 | 1646.3 | 1947.5 | 9.7 | 4.9 | 28.2 | 418 | 239 | 179 | 68 | 21 | 19 | 74 | 225.0 | 2 | 260 | 210 | 64 |
| 3DD2 | H | B | 1.9 | ssRNA | -7.9 | 1508.3 | 718.5 | 789.9 | 10.1 | 5.8 | 19.5 | 159 | 77 | 82 | 23 | 11 | 13 | 87 | -2068.3 | - | - | - | - |
| 3DH3 | AB | EF | 3 | snRNA | -34.9 | 6061.2 | 3057.4 | 3003.8 | 21.5 | 11.7 | 36.6 | 661 | 335 | 326 | 96 | 35 | 42 | 90 | - | 2 | 116 | 79 | 13 |
| 3EPH | A | E | 2.95 | siRNA | -37.7 | 4814.4 | 2297.4 | 2517.1 | 16.9 | 10.7 | 21.5 | 516 | 273 | 243 | 79 | 27 | 41 | 96 | -11470.2 | 4 | 376 | 365 | 56 |
| 3FOZ | B | D | 2.5 | tRNA | -25.8 | 4175.8 | 2077.7 | 2098.1 | 13.3 | 17.5 | 17.9 | 422 | 220 | 202 | 64 | 19 | 33 | 72 | 623.9 | 2 | 293 | 296 | 67 |
| 3LWQ | ABC | DE | 2.68 | tRNA | -29.7 | 5251.9 | 2527.7 | 2724.1 | 17.2 | 11.3 | 20.6 | 569 | 263 | 306 | 84 | 39 | 40 | 40 | -4166.8 | 1 | 305 | 416 | 72 |
| 3MOJ | B | A | 2.9 | tRNA | -9.5 | 1758.4 | 856.7 | 901.7 | 11 | 18.7 | 6.9 | 204 | 86 | 118 | 26 | 15 | 12 | 69 | -7871.8 | 2 | 102 | 143 | 73 |
| 3NVI | AB | E | 2.71 | Miscellaneous | -33.5 | 3782.5 | 1836.4 | 1946.1 | 18.9 | 9.7 | 40.3 | 395 | 185 | 210 | 58 | 23 | 36 | 84 | -3223.6 | 1 | 241 | 293 | 74 |
| 3O3I | X | A | 2.8 | tRNA | -10.2 | 923 | 404.6 | 518.3 | 10.1 | 6.1 | 15.2 | 104 | 59 | 45 | 19 | 5 | 19 | 26 | - | 0 | 74 | 88 | 74 |
| 3O8C | A | C | 2 | tRNA | -17.2 | 1811.8 | 850.7 | 961.1 | 6.7 | 3.1 | 61.7 | 187 | 108 | 79 | 34 | 5 | 11 | 23 | -278.2 | 1 | 94 | 116 | 47 |
| 3RC8 | A | E | 2.9 | mRNA | -12.9 | 1470.6 | 668.1 | 802.5 | 5 | 2.3 | 57.3 | 166 | 91 | 75 | 30 | 6 | 11 | 0 | -6.8 | 0 | 114 | 83 | 50 |
| 3RW6 | AB | FH | 2.3 | Miscellaneous | -18.4 | 5338.1 | 2605 | 2733 | 12.5 | 9.5 | 13.3 | 594 | 285 | 309 | 89 | 56 | 7 | 45 | -11132.6 | 3 | 244 | 443 | 61 |
| 3SNP | AB | CD | 2.8 | rRNA | -25.6 | 5698.8 | 2691.2 | 3007.7 | 7.8 | 4 | 27.2 | 644 | 343 | 301 | 120 | 37 | 29 | 13 | -4964.8 | 0 | 363 | 408 | 58 |
| 3T5N | A | C | 1.79 | tRNA | -11.4 | 1909.5 | 857.1 | 1052.5 | 14.5 | 6.4 | 62.1 | 209 | 121 | 88 | 38 | 6 | 20 | 100 | -1024.7 | 3 | 192 | 174 | 55 |

| | | | | | | | | | | | | | | | | | | | | | | | |
|---|---|---|---|---|---|---|---|---|---|---|---|---|---|---|---|---|---|---|---|---|---|---|---|
| 4ARC | A | B | 2 | tRNA | -32 | 4602.9 | 2216.6 | 2386.2 | 10.1 | 5.9 | 18.7 | 514 | 259 | 255 | 77 | 33 | 24 | 3 | 7959.2 | 1 | 201 | 265 | 47 |
| 4ATO | A | G | 2.2 | tRNA | -12.4 | 2264 | 1103.4 | 1160.6 | 16.3 | 11.3 | 18.2 | 244 | 119 | 125 | 33 | 12 | 26 | 88 | -2527.0 | 2 | 160 | 181 | 73 |
| 4C7O | BD | E | 2.6 | rRNA | -12.5 | 2554.3 | 1194.4 | 1359.9 | 8 | 4.6 | 15.7 | 296 | 147 | 149 | 41 | 22 | 9 | 5 | 2699.3 | 2 | 190 | 246 | 73 |
| 4F3T | A | R | 2.25 | tRNA | -34.4 | 4012.3 | 1766.3 | 2246 | 10.5 | 4.6 | 59.5 | 420 | 232 | 188 | 79 | 14 | 39 | 96 | -7116.6 | 1 | 333 | 352 | 63 |
| 4H5P | AB | E | 2.15 | tRNA | -59.4 | 4482.1 | 2172.3 | 2309.8 | 18.6 | 8.6 | 66.3 | 464 | 253 | 211 | 76 | 14 | 25 | 97 | -4439.8 | 2 | 284 | 449 | 57 |
| 4ILL | AB | CR | 2.48 | crRNA | -37.3 | 7074.1 | 3270.8 | 3803.2 | 25.7 | 12.8 | 42.4 | 757 | 385 | 372 | 114 | 33 | 61 | 83 | - | 1 | 437 | 607 | 67 |
| 4JNG | ABCD | L | 2.12 | Miscellaneous | -131.2 | 11308.9 | 5548.5 | 5760.4 | 25.6 | 12.2 | 58.9 | 1208 | 624 | 584 | 172 | 42 | 69 | 89 | -15264.6 | 11 | 594 | 1006 | 61 |
| 4KR6 | AB | CD | 2.85 | Miscellaneous | -22.1 | 5756 | 2751.9 | 3004.1 | 13.7 | 8.5 | 19.6 | 652 | 321 | 331 | 98 | 44 | 33 | 39 | -14237.3 | 0 | 363 | 420 | 64 |
| 4LGT | A | E | 1.3 | Miscellaneous | -28 | 3375.5 | 1680.9 | 1694.6 | 23.5 | 12.6 | 38.1 | 370 | 184 | 186 | 52 | 17 | 32 | 77 | -2633.6 | 0 | 38 | 1 | 2 |
| 4M59 | AB | CD | 2.46 | tRNA | -56 | 7841.6 | 3664.4 | 4177.2 | 12.4 | 5.9 | 50.7 | 871 | 456 | 415 | 156 | 30 | 25 | 39 | - | 6 | 428 | 730 | 60 |
| 4N0T | A | B | 1.7 | tRNA | -19.9 | 5505.8 | 2648.8 | 2857 | 19.6 | 12.9 | 21.9 | 617 | 310 | 307 | 79 | 30 | 0 | 67 | -11090.9 | 8 | 352 | 671 | 72 |
| 4NGC | A | B | 2.1 | Miscellaneous | -12.2 | 1021.1 | 440.3 | 580.8 | 6 | 2.9 | 19.4 | 123 | 71 | 52 | 27 | 7 | 0 | 96 | -1132.5 | 1 | 96 | 118 | 56 |
| Low | | | | | | | | | | | | | | | | | | | | | | | |
| 1DFU | P | MN | 1.8 | rRNA | -20.2 | 1687.9 | 828.2 | 859.7 | 19.5 | 11.8 | 17.2 | 172 | 84 | 88 | 26 | 19 | 19 | 73 | -3741.2 | 0 | 86 | 78 | 54 |
| 1DI2 | AB | CDEG | 1.9 | rRNA | -23.9 | 2255.6 | 1178.4 | 1077.3 | 14 | 12 | 14.8 | 237 | 107 | 130 | 32 | 22 | 9 | 97 | -4985.1 | 0 | 149 | 139 | 60 |
| 1F7Y | A | B | 2.8 | tRNA | -27.6 | 2426.6 | 1220.2 | 1206.5 | 13.3 | 13.2 | 15.3 | 265 | 130 | 135 | 33 | 22 | 19 | 67 | -6690.5 | - | - | - | - |
| 1G1X | ABC | DE | 2.6 | ssRNA | -45.4 | 4944.1 | 2426.4 | 2517.7 | 16.2 | 17.9 | 20.5 | 546 | 270 | 276 | 70 | 47 | 35 | 61 | -18347.4 | 1 | 252 | 223 | 58 |
| 1I6U | A | C | 2.6 | Miscellaneous | -11.6 | 1801.5 | 885.1 | 916.4 | 11.6 | 14 | 14.6 | 213 | 106 | 107 | 30 | 19 | 16 | 90 | -6090.4 | 0 | 20 | 27 | 17 |
| 1JID | A | B | 1.8 | crRNA | -13.6 | 1362.1 | 665.8 | 696.4 | 9.5 | 13 | 12.4 | 142 | 69 | 73 | 25 | 9 | 12 | 100 | -4365.2 | 2 | 121 | 56 | 40 |
| 1L9A | A | B | 2.9 | dsRNA | -25.8 | 2210.2 | 1074.4 | 1135.7 | 18.5 | 5.3 | 8.8 | 236 | 107 | 129 | 27 | 21 | 22 | 96 | -13982.7 | 0 | 185 | 126 | 63 |
| 1MJI | AB | CD | 2.5 | dsRNA | -36.6 | 5602.4 | 2776.6 | 2825.8 | 13 | 24.4 | 20.5 | 611 | 289 | 322 | 81 | 47 | 37 | 73 | -16485.6 | 0 | 23 | 28 | 7 |
| 1MZP | A | B | 2.65 | dsRNA | -26.5 | 2883.3 | 1474 | 1409.4 | 12.5 | 15.6 | 16 | 296 | 139 | 157 | 37 | 26 | 31 | 97 | -14273.9 | 0 | 11 | 3 | 5 |
| 1N35 | A | BC | 2.5 | dsRNA | -31.6 | 3243.9 | 1557.6 | 1686.3 | 3.3 | 55.3 | 6.9 | 355 | 189 | 166 | 71 | 13 | 17 | 93 | -1922.5 | 0 | 200 | 183 | 49 |
| 1QU3 | A | T | 2.9 | dsRNA | -36.9 | 4847.5 | 2468.5 | 2379 | 6.3 | 18.7 | 10.2 | 543 | 270 | 273 | 72 | 37 | 32 | 18 | 18109.1 | 0 | 350 | 346 | 65 |
| 1RPU | AB | CD | 2.5 | dsRNA | -36 | 3917.8 | 1877.5 | 2040.4 | 13.1 | 24.6 | 21 | 433 | 214 | 219 | 66 | 27 | 40 | - | -5572.0 | 2 | 218 | 254 | 59 |
| 1S03 | H | A | 2.7 | dsRNA | -16 | 1743.1 | 847.8 | 895.3 | 11.1 | 10.4 | 12 | 204 | 101 | 103 | 29 | 16 | 10 | 86 | -4105.7 | 0 | 110 | 133 | 72 |
| 1SER | AB | T | 2.9 | tRNA | -18.7 | 2259 | 1121.3 | 1137.7 | 3.4 | 10.3 | 5.4 | 265 | 126 | 139 | 40 | 17 | 11 | 20 | - | 0 | 66 | 70 | 30 |
| 1WSU | ABC | EFG | 2.3 | dsRNA | -32.2 | 2631.4 | 1283.2 | 1348.3 | 5.1 | 11.4 | 7.6 | 268 | 125 | 143 | 39 | 18 | 27 | 46 | -4986.2 | 2 | 214 | 123 | 51 |
| 1Y39 | A | C | 2.8 | Miscellaneous | -18.1 | 1988.8 | 993.1 | 995.7 | 19.4 | 12.1 | 17.6 | 212 | 97 | 115 | 30 | 19 | 16 | 83 | -3276.1 | 0 | 192 | 189 | 87 |
| 2AZ2 | AB | CD | 2.6 | dsRNA | -20.6 | 2043 | 976 | 1067 | 12.7 | 15.9 | 16.6 | 214 | 115 | 99 | 30 | 14 | 13 | 17 | -1657.3 | 0 | 18 | 20 | 13 |
| 2BTE | A | B | 2.9 | Miscellaneous | -28.1 | 3430.9 | 1689.3 | 1741.6 | 4.3 | 12.8 | 7 | 379 | 184 | 195 | 63 | 29 | 21 | 0 | 6855.2 | 0 | 168 | 190 | 46 |
| 2HW8 | A | B | 2.1 | dsRNA | -34.4 | 2334.7 | 1121.6 | 1213.1 | 9.5 | 18.9 | 14.6 | 271 | 142 | 129 | 35 | 21 | 16 | 34 | -4690.2 | 0 | 17 | 14 | 9 |
| 2J0S | A | E | 2.21 | dsRNA | -21.3 | 1310.4 | 538.8 | 771.6 | 3.1 | 47.4 | 7.3 | 150 | 79 | 71 | 28 | 6 | 19 | 0 | -505.5 | 0 | 167 | 97 | 71 |
| 2JLU | A | C | 2.04 | dsRNA | -17.8 | 1925.2 | 846 | 1079.2 | 40 | 64.8 | 9.2 | 209 | 121 | 88 | 39 | 7 | 14 | 95 | - | 0 | 158 | 136 | 67 |
| 2V3C | AC | M | 2.5 | dsRNA | -43.7 | 5181.3 | 2547.9 | 2633.4 | 9.3 | 16.9 | 13.8 | 592 | 280 | 312 | 82 | 50 | 0 | 54 | -19704.8 | 1 | 350 | 321 | 62 |
| 2XLI | A | B | 2.33 | dsRNA | -11.9 | 1566.3 | 830.1 | 736.2 | 8.4 | 25.6 | 14.1 | 145 | 76 | 69 | 26 | 12 | 16 | 65 | - | 2 | 116 | 101 | 35 |
| 2ZH9 | A | B | 2.9 | rRNA | -18.8 | 2425.2 | 1141.8 | 1283.4 | 4.8 | 21 | 8.9 | 275 | 148 | 127 | 42 | 16 | 13 | 21 | -2174.3 | 0 | 182 | 198 | 67 |
| 2ZKO | AB | CD | 1.7 | rRNA | -29 | 2465.8 | 1238.1 | 1227.6 | 14.3 | 15.6 | 17.6 | 264 | 128 | 136 | 34 | 20 | 11 | 56 | -4146.9 | 0 | 161 | 173 | 65 |
| 3AVW | A | GT | 2.6 | rRNA | -30.3 | 3382.2 | 1613.7 | 1768.4 | 3 | 43.3 | 6.3 | 374 | 195 | 179 | 73 | 20 | 14 | 7 | - | 0 | 182 | 183 | 49 |
| 3EQT | AB | CD | 2 | Miscellaneous | -20 | 2705.3 | 1215.9 | 1489.4 | 7.3 | 48.1 | 15.9 | 285 | 141 | 144 | 48 | 16 | 20 | 81 | -4117.2 | 0 | 208 | 253 | 74 |
| 3FTF | A | CD | 2.8 | rRNA | -19 | 1693.3 | 841.6 | 851.7 | 6.8 | 10.8 | 9.1 | 179 | 89 | 90 | 28 | 18 | 5 | 96 | -5610.3 | 0 | 93 | 77 | 54 |
| 3H5X | A | PT | 1.77 | rRNA | -35.6 | 3095.8 | 1458.2 | 1637.6 | 6.7 | 48.1 | 13.9 | 325 | 179 | 146 | 65 | 16 | 21 | 83 | -1199.8 | 0 | 185 | 94 | 14 |
| 3NL0 | A | BC | 2.6 | dsRNA | -26.5 | 2720.6 | 1238.5 | 1482 | 5.9 | 54.7 | 12.9 | 312 | 169 | 143 | 59 | 13 | 19 | 69 | 479.8 | 0 | 235 | 193 | 63 |
| 3R2D | ABJK | RS | 2.2 | tRNA | -31.6 | 2090.3 | 1014.8 | 1075.5 | 4.5 | 25.9 | 8.5 | 224 | 118 | 106 | 40 | 14 | 11 | 90 | -6266.9 | 0 | 148 | 77 | 60 |

| | | | | | | | | | | | | | | | | | | | | | | | |
|---|---|---|---|---|---|---|---|---|---|---|---|---|---|---|---|---|---|---|---|---|---|---|---|
| 3TMI | A | BC | 2.7 | siRNA | -32.9 | 2828.7 | 1300.5 | 1528.2 | 4 | 33.3 | 8.2 | 332 | 181 | 151 | 61 | 21 | 14 | 98 | - | 1 | 211 | 200 | 56 |
| 3VJR | A | B | 2.4 | tRNA | -12.8 | 1354.3 | 689.9 | 664.3 | 7.5 | 10 | 9.4 | 144 | 63 | 81 | 20 | 15 | 11 | 55 | -1976.3 | 0 | 82 | 75 | 75 |
| 3VYY | AB | CDEF | 2.9 | mRNA | -35.2 | 3073.9 | 1550.1 | 1523.9 | 13.3 | 21.3 | 19.5 | 349 | 168 | 181 | 40 | 25 | 3 | 50 | -6151.8 | 0 | 179 | 217 | 68 |
| 3WBM | ABCD | XY | 2 | dsRNA | -31.5 | 2348.8 | 1173.5 | 1175.3 | 6.5 | 12.6 | 9.3 | 244 | 115 | 129 | 32 | 23 | 18 | 100 | -20163.4 | 0 | 149 | 76 | 51 |
| 3ZC0 | AB | M | 2.98 | mRNA | -9.5 | 814 | 414.7 | 399.4 | 2 | 12.4 | 3.6 | 84 | 39 | 45 | 17 | 6 | 0 | 27 | - | 0 | 41 | 12 | 15 |
| 4E78 | A | PT | 2.9 | mRNA | -15.7 | 1674.8 | 783 | 891.8 | 3.4 | 47 | 7.3 | 193 | 108 | 85 | 43 | 8 | 8 | 98 | -2092.3 | 0 | 93 | 94 | 44 |
| 4FVU | A | BC | 2.91 | tRNA | -10 | 1240.1 | 574.5 | 665.7 | 5.2 | 21.4 | 9.6 | 139 | 72 | 67 | 24 | 9 | 10 | 12 | - | 1 | 88 | 96 | 58 |
| 4GHA | ACEG | IJ | 2.5 | mRNA | -34.5 | 3690.9 | 1774 | 1916.9 | 7.2 | 41.8 | 14.5 | 395 | 207 | 188 | 71 | 23 | 19 | 65 | -7419.2 | 0 | 216 | 240 | 60 |
| 4IG8 | A | BC | 2.7 | Miscellaneous | -22.3 | 2914 | 1415.2 | 1498.8 | 8.4 | 22.9 | 14.3 | 316 | 166 | 150 | 46 | 20 | 23 | 87 | -5071.9 | 0 | 126 | 123 | 39 |
| 4K4T | A | BC | 2.75 | Miscellaneous | -34.1 | 3724.2 | 1758.1 | 1966.1 | 8.2 | 32.8 | 15.7 | 426 | 227 | 199 | 72 | 21 | 22 | 78 | 119.2 | 0 | 242 | 266 | 58 |
| 4K50 | A | BC | 2.93 | dsRNA | -37.1 | 4061.9 | 1916.1 | 2145.7 | 8.9 | 23.8 | 15.4 | 450 | 242 | 208 | 75 | 23 | 24 | 77 | -762.6 | 0 | 267 | 279 | 64 |
| 4OOG | C | D | 2.5 | Miscellaneous | -26.8 | 3053.4 | 1504 | 1549.4 | 9.8 | 23.4 | 16.1 | 326 | 160 | 166 | 45 | 22 | 5 | 73 | -4854.8 | 2 | 200 | 227 | 73 |

**Table S3.** Numbers of different types of RNAs in three classes.

| type | ssRNA | dsRNA | mRNA | tRNA | rRNA | Other | All |
|---|---|---|---|---|---|---|---|
| High class | 6 | 1 | 13 | 0 | 4 | 9 | 33 |
| Medium class | 3 | 2 | 4 | 25 | 4 | 23 | 61 |
| Low class | 1 | 17 | 4 | 5 | 7 | 9 | 43 |

**Table S4.** Detailed information of the AAC and AAP in each class.

| Type | Ala | Phe | Gly | Ile | Leu | Met | Pro | Val | Asp | Glu | Lys | Arg | Cys | His | Asn | Gln | Ser | Thr | Trp | Tyr |
|---|---|---|---|---|---|---|---|---|---|---|---|---|---|---|---|---|---|---|---|---|
| High | | | | | | | | | | | | | | | | | | | | |
| AAC | 3.62 | 4.94 | 7.64 | 5.86 | 6.39 | 1.45 | 2.96 | 4.48 | 4.87 | 5.33 | 12.18 | 10.34 | 1.053 | 4.28 | 5.33 | 5.13 | 4.74 | 3.29 | 0.00 | 0.06 |
| AAP | -0.29 | 0.77 | 0.04 | 0.44 | 0.12 | 0.20 | -0.42 | 0.19 | -0.53 | -0.84 | 0.13 | 0.42 | 0.96 | 0.20 | -0.06 | -0.03 | -0.18 | -0.46 | 0.51 | 0.84 |
| Medium | | | | | | | | | | | | | | | | | | | | |
| AAC | 5.45 | 3.37 | 5.50 | 3.34 | 5.24 | 1.75 | 4.61 | 4.71 | 4.69 | 5.14 | 10.99 | 13.91 | 0.81 | 3.17 | 6.51 | 3.60 | 7.02 | 5.04 | 0.01 | 0.04 |
| AAP | -0.08 | 0.50 | -0.30 | 0.17 | 0.01 | 0.20 | -0.21 | 0.10 | -0.55 | -0.86 | 0.07 | 0.61 | 0.40 | 0.38 | 0.38 | -0.37 | 0.12 | -0.04 | 0.27 | 0.47 |
| Low | | | | | | | | | | | | | | | | | | | | |
| AAC | 4.42 | 2.05 | 7.45 | 3.49 | 4.93 | 1.90 | 4.52 | 4.42 | 5.29 | 4.88 | 12.63 | 13.92 | 1.18 | 2.52 | 4.88 | 4.16 | 7.04 | 6.06 | 0.01 | 0.03 |
| AAP | -0.22 | -0.16 | 0.06 | 0.17 | -0.11 | 0.24 | -0.30 | -0.02 | -0.38 | -0.93 | 0.16 | 0.54 | 0.90 | 0.21 | 0.09 | 0.02 | 0.19 | 0.19 | 0.29 | 0.17 |

**Table S5.** Detailed information of the SSC and SSP in each class.

| Type | α-helix | β-strand | $3_{10}$-helix | Turn | coil | Bridge | non-regular[a] |
|---|---|---|---|---|---|---|---|
| High | | | | | | | |
| SSC | 0.30 | 0.33 | 0.06 | 0.16 | 0.15 | 0.00 | 0.37 |
| SSP | -0.16 | 0.67 | 0.67 | -0.22 | -0.26 | -4.47 | -0.26 |
| Medium | | | | | | | |
| SSC | 0.32 | 0.14 | 0.06 | 0.25 | 0.21 | 0.02 | 0.54 |
| SSP | -0.16 | 0.14 | 0.48 | 0.09 | -0.07 | 0.48 | 0.07 |
| Low | | | | | | | |
| SSC | 0.36 | 0.17 | 0.04 | 0.20 | 0.21 | 0.01 | 0.47 |
| SSP | -0.05 | 0.35 | 0.01 | -0.11 | -0.07 | 0.45 | -0.07 |

[a] $3_{10}$-helix, turn, coil and bridge are together deemed as the non-regular regions.

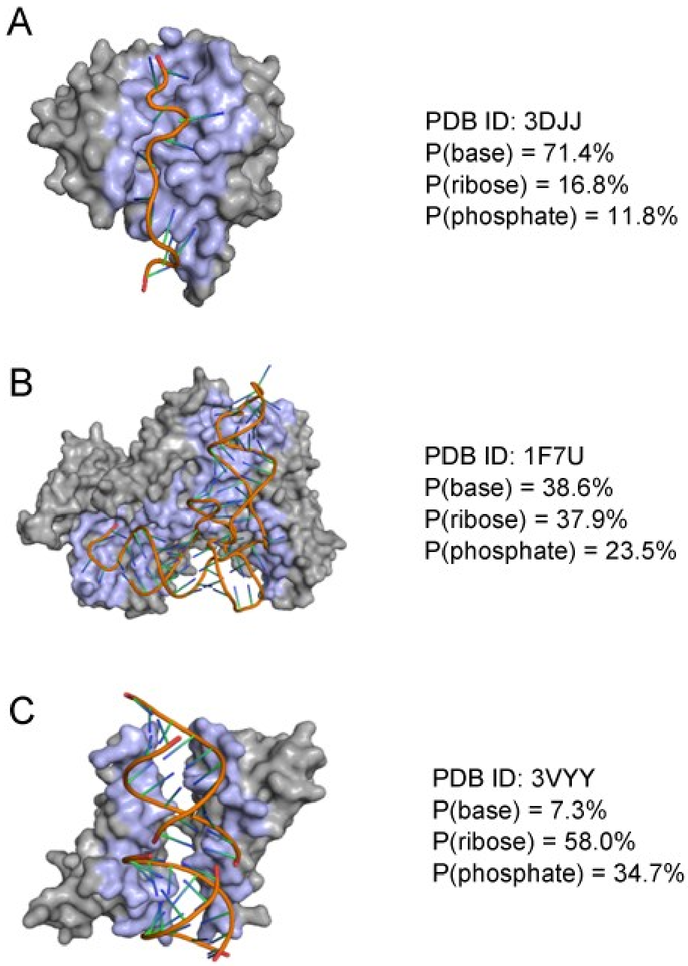


**Figure S1.** The 3D structures of three representative complexes in High, Medium and Low class, respectively. The protein interface region is colored lightblue.
